# The Extracellular Milieu of *Toxoplasma*'s Lytic Cycle Drives Lab Adaptation, Primarily by Transcriptional Reprogramming

**DOI:** 10.1128/mSystems.01196-21

**Published:** 2021-12-07

**Authors:** Vincent A. Primo, Yasaman Rezvani, Andrew Farrell, Connor Q. Murphy, Jingjing Lou, Amir Vajdi, Gabor T. Marth, Kourosh Zarringhalam, Marc-Jan Gubbels

**Affiliations:** a Department of Biology, Boston College, Chestnut Hill, Massachusetts, USA; b Department of Mathematics, University of Massachusetts Boston, Boston, Massachusetts, USA; c Department of Human Genetics and USTAR Center for Genetic Discovery, Eccles Institute of Human Genetics, University of Utah School of Medicine, Salt Lake City, Utah, USA; d Department of Computer Science, University of Massachusetts Boston, Boston, Massachusetts, USA; University of Hawaii at Manoa

**Keywords:** *Toxoplasma*, lab adaptation, serial passaging, experimental evolution, evolve and resequencing, regression analysis, genotype-phenotype correlation

## Abstract

Evolve and resequencing (E&R) was applied to lab adaptation of Toxoplasma gondii for over 1,500 generations with the goal of mapping host-independent *in vitro* virulence traits. Phenotypic assessments of steps across the lytic cycle revealed that only traits needed in the extracellular milieu evolved. Nonsynonymous single-nucleotide polymorphisms (SNPs) in only one gene, a P4 flippase, fixated across two different evolving populations, whereas dramatic changes in the transcriptional signature of extracellular parasites were identified. Newly developed computational tools correlated phenotypes evolving at different rates with specific transcriptomic changes. A set of 300 phenotype-associated genes was mapped, of which nearly 50% is annotated as hypothetical. Validation of a select number of genes by knockouts confirmed their role in lab adaptation and highlights novel mechanisms underlying *in vitro* virulence traits. Further analyses of differentially expressed genes revealed the development of a “pro-tachyzoite” profile as well as the upregulation of the fatty acid biosynthesis (FASII) pathway. The latter aligned with the P4 flippase SNP and aligned with a low abundance of medium-chain fatty acids at low passage, indicating this is a limiting factor in extracellular parasites. In addition, partial overlap with the bradyzoite differentiation transcriptome in extracellular parasites indicated that stress pathways are involved in both situations. This was reflected in the partial overlap between the assembled ApiAP2 and Myb transcription factor network underlying the adapting extracellular state with the bradyzoite differentiation program. Overall, E&R is a new genomic tool successfully applied to map the development of polygenic traits underlying *in vitro* virulence of T. gondii.

**IMPORTANCE** It has been well established that prolonged *in vitro* cultivation of Toxoplasma gondii augments progression of the lytic cycle. This lab adaptation results in increased capacities to divide, migrate, and survive outside a host cell, all of which are considered host-independent virulence factors. However, the mechanistic basis underlying these enhanced virulence features is unknown. Here, E&R was utilized to empirically characterize the phenotypic, genomic, and transcriptomic changes in the non-lab-adapted strain, GT1, during 2.5 years of lab adaptation. This identified the shutdown of stage differentiation and upregulation of lipid biosynthetic pathways as the key processes being modulated. Furthermore, lab adaptation was primarily driven by transcriptional reprogramming, which rejected the starting hypothesis that genetic mutations would drive lab adaptation. Overall, the work empirically shows that lab adaptation augments T. gondii’s *in vitro* virulence by transcriptional reprogramming and that E&R is a powerful new tool to map multigenic traits.

## INTRODUCTION

Toxoplasma gondii is an apicomplexan parasite able to infect virtually any warm-blooded animal and causes opportunistic infections in humans (1). Disease manifestations are typically mild. However, severe and life-threatening illness is associated with low immunocompetence or is the result of specific combinations of parasite and host genotypes that lead to failures in the immune response. Disease severity is also defined by additional host-independent virulence traits, which constitute aspects of the lytic cycle, such as replication rate, host cell invasion capacity, tissue transmigration efficiency, and enhanced survival in the extracellular environment (2). Identifying the genetic basis of these host-independent virulence traits will provide insights in universal virulence mechanisms.

During *in vitro* lab adaptation (i.e., from *in vivo* isolation to continuous *in vitro* cultivation), many of the general, host-independent virulence traits become enhanced (2, 3). The lab-adapted RH model strain produces >5-fold larger plaques *in vitro* (i.e., *in vitro* virulence) compared to non-lab-adapted strains of the same type I genotype (2). In particular, RH’s replication rate and extracellular viability are superior to the non-lab-adapted GT1 strain (2, 4). Efforts to identify the genetic basis for these phenotypic differences identified 1,394 single-nucleotide polymorphisms (SNPs) between RH and GT1, of which 133 caused amino acid changes and 54 were insertions/deletions within coding regions (5). Since experimental validation by allele swapping did not reveal major drivers of *in vitro* virulence (5), we hypothesized that the genetic basis is a combination of alleles (i.e., epistasis). However, the limited chronological record of RH’s *in vitro* history prevented dissection of the genotype-phenotype relationship.

Evolve and resequencing (E&R) is a universal tool to dissect the genetic basis of adaptive or selective processes as it permits real-time investigation of genetic factors underlying experimental evolution (6, 7). The most famous is Lenski’s long-term experimental evolution (LTEE) experiment of Escherichia coli (8), which identified alleles and expression profiles responsible for adaptation (9–13). We applied E&R to the non-lab-adapted T. gondii GT1 strain to both establish a chronological record of T. gondii lab adaptation and identify the genes and mechanisms underlying T. gondii’s host-independent *in vitro* virulence traits. Over 2 years of lab adaptation, we observed a steady phenotype adaptation rate. However, only one gene with a nonsynonymous SNP fixated in evolving populations. On the other hand, significant transcriptional changes in the expression of ∼1,000 genes accompanied evolution. These gene expression changes were almost exclusively restricted to extracellular parasites. The transcriptional signature revealed shutdowns of differentiation exits, resulting in a “pro-tachyzoite” profile as well as upregulation of the fatty acid (FA) biosynthesis pathway, which aligned with the limited medium-chain FA abundance at low passage. Regression and clustering analysis of differentially expressed genes (DEGs) identified 300 genes that strongly correlated with changes in phenotypes. Many of these genes are hypothetical, but experimental validation of select genes confirmed their roles in GT1’s evolution. Finally, the transcriptional data were used to assemble a transcriptional network, which consolidated the defined roles of several characterized transcription factors (14, 15), next to identifying a Myb and several AP2 factors specifically associated with (adaptation to) the extracellular environment. Taken together, we successfully applied lab adaptation and E&R as a tool to identify a rich set of 300 host-independent virulence factors.

## RESULTS

### Lab adaptation of GT1 results in enhanced *in vitro* virulence.

**FIG 1:**
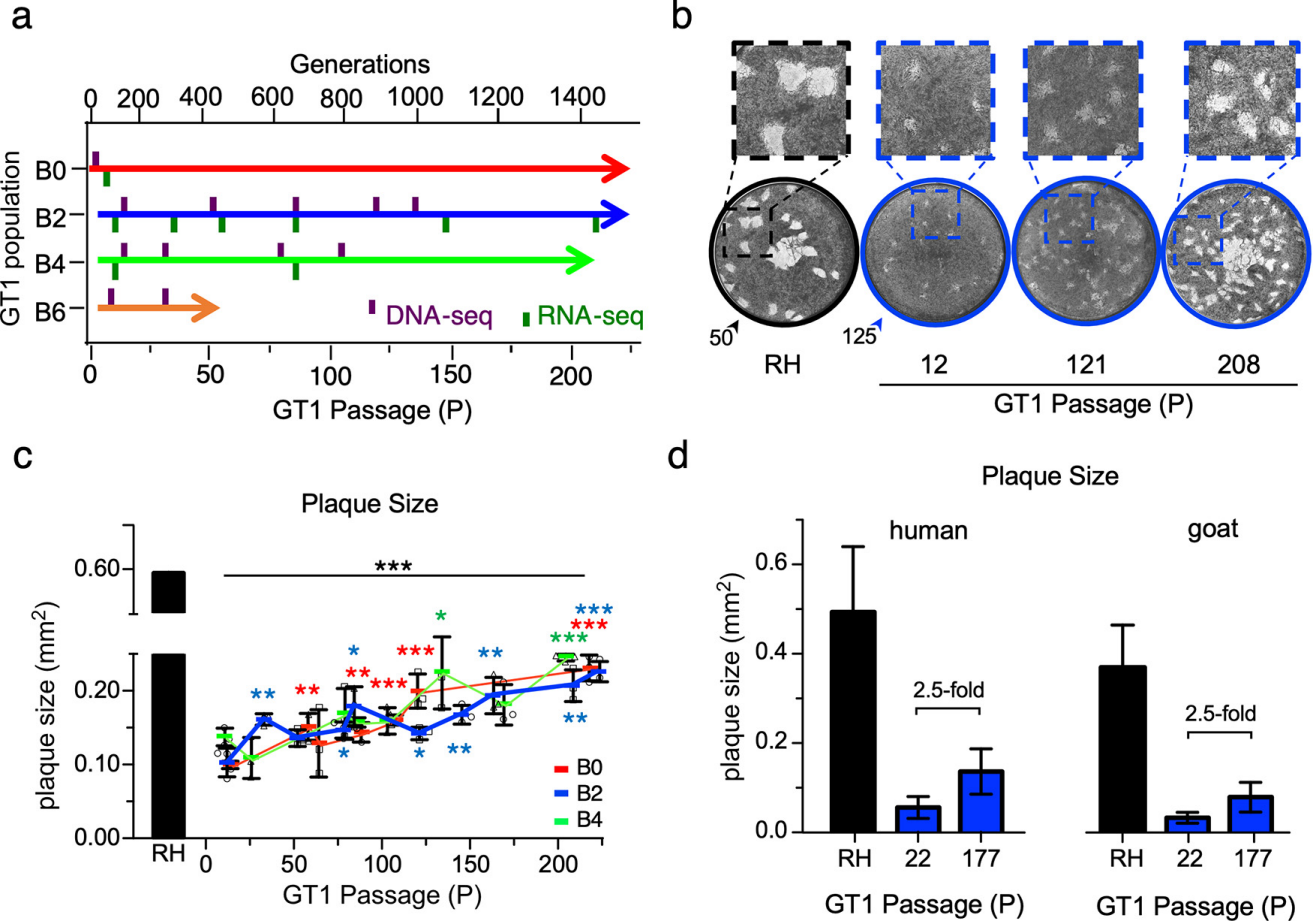
Augmented *in vitro* virulence of GT1 following lab adaptation. (a) Experimental timeline of parallel GT1 lab adaptation experiments and DNA-Seq/RNA-Seq time points drawn to scale. B0 represents the polyclonal starting line, whereas B2, B4, and B6 represent distinct clones generated from B0 at the lowest possible passage. (b) Representative images of an 11-day plaque assay on HFF host cells. Arrowheads indicate the number of RH or GT1 (B2) parasites inoculated. (c) Quantification of plaque area following 11-day plaque assays with RH or GT1 (B0 [red], B2 [blue], B4 [green]; color coded as in panel a) parasites. Black asterisks indicate the *P* value of the indicated GT1 passages relative to RH; colored asterisks corresponding with the lineages indicate the *P* value of the indicated GT1 passage relative to the respective population’s earliest passage. *, *P* ≤ 0.05; **, *P* ≤ 0.01; ***, *P* ≤ 0.001. Colored blocks indicate mean of ≥3 biological replicates for B0, B2, and B4 (shown as squares, circles, and triangles, respectively), with error bars representing SD. One biological replicate is the mean quantification of ≥25 plaques. (d) Plaque assay of RH or GT1 (B2) using both human-derived (HFF; human foreskin fibroblasts) and goat-derived (GSF; goat skeletal fibroblasts) host cells; *n* = 1. The error bar indicates standard deviations (SD) from ≥20 plaques across two technical replicates.

To ascertain a homogenous starting population, we first established independent clones from a limited-passage, cryopreserved GT1 strain used to establish T. gondii’s reference genome (4). We propagated the original uncloned GT1 strain (designated B0) and three independent clones (B2, B4, and B6) in immortalized human foreskin fibroblasts (HFFs). Five percent of the parasite culture was passaged every 2 to 3 days for up to 223 serial passages (P), spanning ∼2 years and about ∼1,500 parasite generations (Fig. 1a). As a proxy for GT1’s lab adaptation, we performed plaque assays along the evolutionary path and observed a steady increase (Fig. 1b and c): B0 increased 2.30-fold (*P* ≤ 2.3 × 10^−4^), B2 increased 2.19-fold (*P* ≤ 8.9 × 10^−4^), and B4 increased 1.86-fold (*P* ≤ 1.1 × 10^−4^). However, plaque size of the lab-adapted RH strain, used as the gold standard throughout our experiments, remained 2.31- to 2.62-fold larger than all >P200 GT1 populations (*P* ≤ 6.13 × 10^−5^), indicating that after 2 years of *in vitro* evolution, GT1 has not yet reached the adaptation level of RH (Fig. 1c).

Because RH and GT1 strains were isolated from different species, human and goat, respectively, we tested whether the difference in *in vitro* virulence correlated with the original host. Comparison of plaque sizes of RH, B2-P22, and B2-P177 in primary HFFs versus goat skeletal fibroblasts (GSF) revealed that lab adaptation was host species independent (Fig. 1d).

### Lab adaptation of GT1 results in enhanced extracellular survival and invasion capacities.

**FIG 2:**
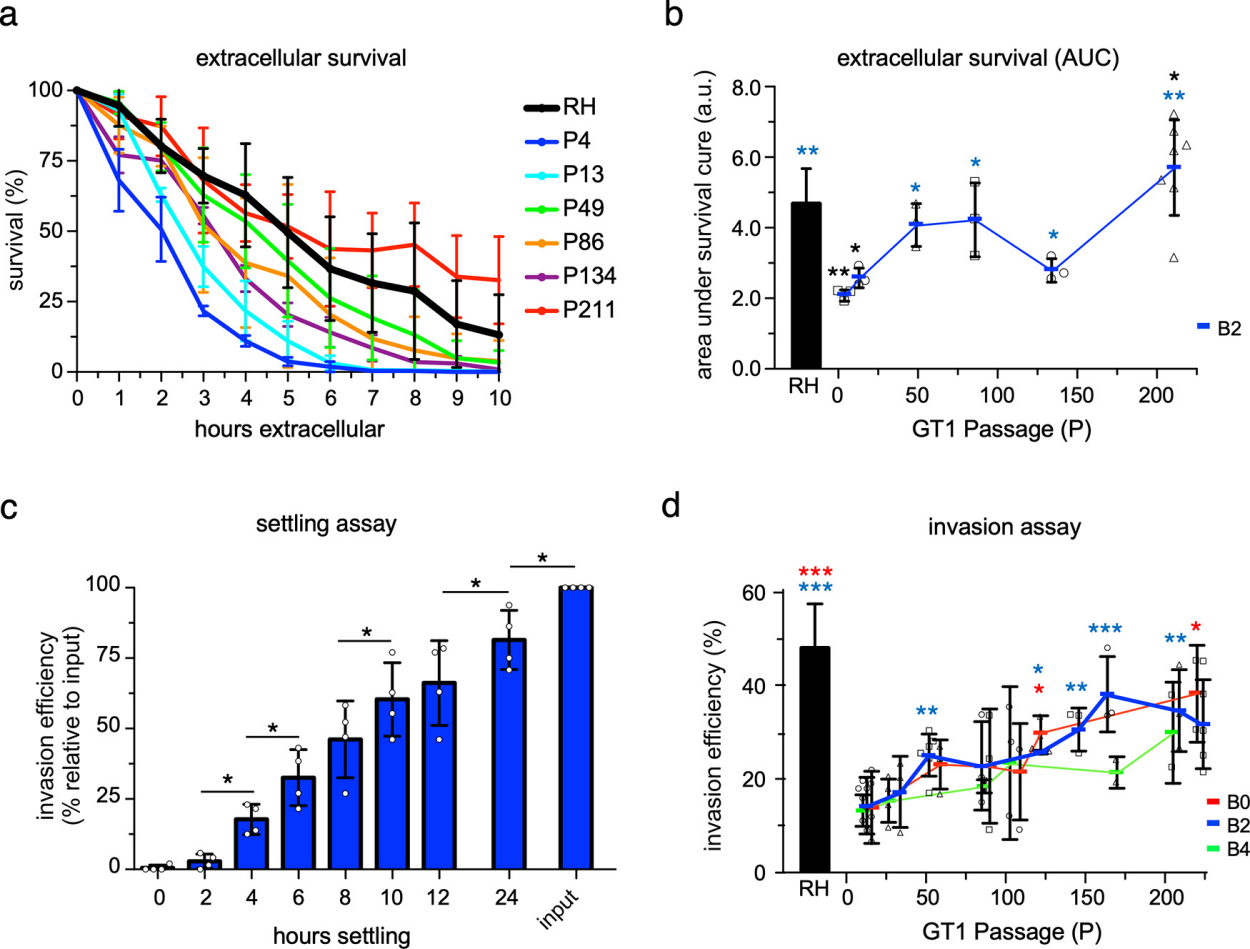
Lab adaptation augments virulence traits of the extracellular milieu of the lytic cycle. (a) Mechanically released RH and GT1 parasites (B2) were incubated without host cells for 0 to 10 h, and survivability was measured hourly by plaque assay. Colored block indicates mean of ≥3 biological replicates with SD plotted; each biological replicate comprises 2 technical replicates. (b) Plot of the area under the survival curves shown in panel a. Blue block indicates mean of ≥3 biological replicates (shown as squares, circles, and triangles) with error bars representing SD. (c) Mechanically egressed RH parasites were allowed to settle by gravity onto host cells for the times indicated before washing away extracellular parasites and analyzing invasion efficiency by plaque assay; data are normalized to an unwashed, total inoculum control (input). The mean from 4 biological replicates (circles) with SD is plotted; each biological replicate comprises 2 technical replicates. (d) Invasion efficiency, i.e., the quantification of the total number of plaques formed relative to the input following mechanical egress of RH or GT1 (B0, B2, and B4) parasites. Horizontal lines indicate mean of biological replicates for B0 (*n* = ≥3), B2 (*n* = ≥3), B4 (*n* = 2), shown as squares, circles, and triangles, respectively, with error bars representing SD. For all panels, black asterisks indicate the *P* value of the indicated GT1 passage relative to RH; colored asterisks indicate the *P* value of the indicated GT1 passage relative to the respective population.

We performed functional assays to identify which step(s) in the lytic cycle contributed to the plaque size increase. We first tested extracellular survival capacity. GT1 parasites of various passages were subjected to extracellular conditions for 0 to 10 h, and viability of the population was assessed hourly by plaque assay, resulting in survival curves (Fig. 2a). The lethal time to kill 50% of the input population (LT_50_) was only 2 h for B2-P4 but increased to 5 h for B2-P211 (*P* ≤ 1.3 × 10^−4^) (Fig. 2a). The area under these survival curves indicated that B2’s overall extracellular viability during the 10-h exposure increased 2.21-fold by B2-P211 relative to B2-P4 (*P* ≤ 2.5 × 10^−3^) (Fig. 2b). The LT_50_ of B2 was already similar to the well-adapted RH reference strain by B2-P50, suggesting that this trait is adapting rapidly.

We wondered how lab adaptation induced such a strong selection pressure on extracellular parasites. We reasoned that upon passaging, the inoculated parasites might spend significant time extracellularly while settling by gravity onto the new host monolayer. To test this hypothesis, we determined the settling kinetics by plaque assay using RH parasites. Indeed, only 50% of RH parasites successfully infected a new T25 host cell monolayer after 8 h, and not even all parasites with plaque-forming capacity had settled after 24 h (Fig. 2c). Thus, extracellular survival is a strong selection pressure during our *in vitro* lab adaptation protocol.

Next, we tested host cell invasion efficiency, measured as the number of plaques relative to inoculum size. Time spent extracellularly was standardized by mechanically releasing the parasites from their host cells by needle passage. All GT1 populations showed a steady increase in invasion efficiency between the first and last measuring point: B0 increased 2.76-fold (*P* ≤ 0.03), B2 increased 2.24-fold (*P* ≤ 0.003), and B4 increased 2.27-fold (*n* = 2; no statistics performed) (Fig. 2d). Interestingly, invasion efficiency of RH remained 2.24- to 2.77-fold larger than that of all >P200 GT1 populations (*P* ≤ 0.007), suggesting that continued lab adaptation of GT1 will result in a continued rise of this virulence trait (Fig. 2d). Hence, GT1’s invasion capacity is a lab-adaptive virulence trait.

We assessed the replication rate by enumerating the parasites per vacuole after 24 h of replication. We observed only very minor shifts in replication efficiency or doubling rates of B2 or B4 populations during lab adaptation (see Fig. S1a to c in the supplemental material). To complete the lytic cycle, egress efficiency was assessed on B2-P12, B2-P83, and B2-P211 by triggering with Ca^2+^ ionophore A23187 or ethanol (Fig. S1d). We observed no significant differences in egress capacity. Thus, neither replication rate nor egress efficiency is a lab-adaptive trait. In conclusion, traits corresponding to the extracellular milieu of the lytic cycle are positively selected for, while traits associated with intracellular development are not.

10.1128/mSystems.01196-21.2 FIG S1 Virulence traits of the intracellular milieu are not affected by lab-adaptation. (a and b) Replication assay of RH and GT1 populations of B2 (a) or B4 (b) parasites at the indicated timepoints. The number of parasites per vacuole in 100 random vacuoles was quantified 24-h postinfection by IFA. Mean of ≥3 biological replicates with SD is plotted. Black asterisks indicate the *P* value of the indicated GT1 passages relative to RH; white asterisks indicate *P* value of the indicated GT1 passage relative to the respective population’s earliest passage (GT1-P16); *, *P* ≤ 0.05; **, *P* ≤ 0.01; ***, *P* ≤ 0.001. (c) Same dataset as in panel a and b represented as average number of parasite duplications during the first 24 h of inoculation. Colored blocks indicate mean of ≥3 biological replicates for B2 and B4 (shown as circles and squares, respectively) with sizeable error bars representing SD. (d) Thirty-hour intracellular parasites were treated for five minutes with either control (DMSO), calcium ionophore (A23187), or ethanol (EtOH). The number of egressed and nonegressed vacuoles were enumerated by IFA. Mean of 3 biological replicates (shown as squares, circles, and triangles) is plotted. Download FIG S1, TIF file, 0.4 MB. Copyright © 2021 Primo et al. 2021 Primo et al. https://creativecommons.org/licenses/by/4.0/ This content is distributed under the terms of the Creative Commons Attribution 4.0 International license.

### WGS identified P4-flippase as a candidate polymorphic virulence factor.

To track genomic mutations, we performed whole-genome sequencing (WGS) on the GT1 populations at several passages during evolution (Fig. 1a). We first assessed the clonality of the three clonally derived populations (B2-P15, B4-P15, and B6-P10) and compared them to the polyclonal parent population (B0-P4). No polymorphisms were identified between these low-passage-number strains, indicating strong clonality within all of our starting GT1 populations.

**FIG 3:**
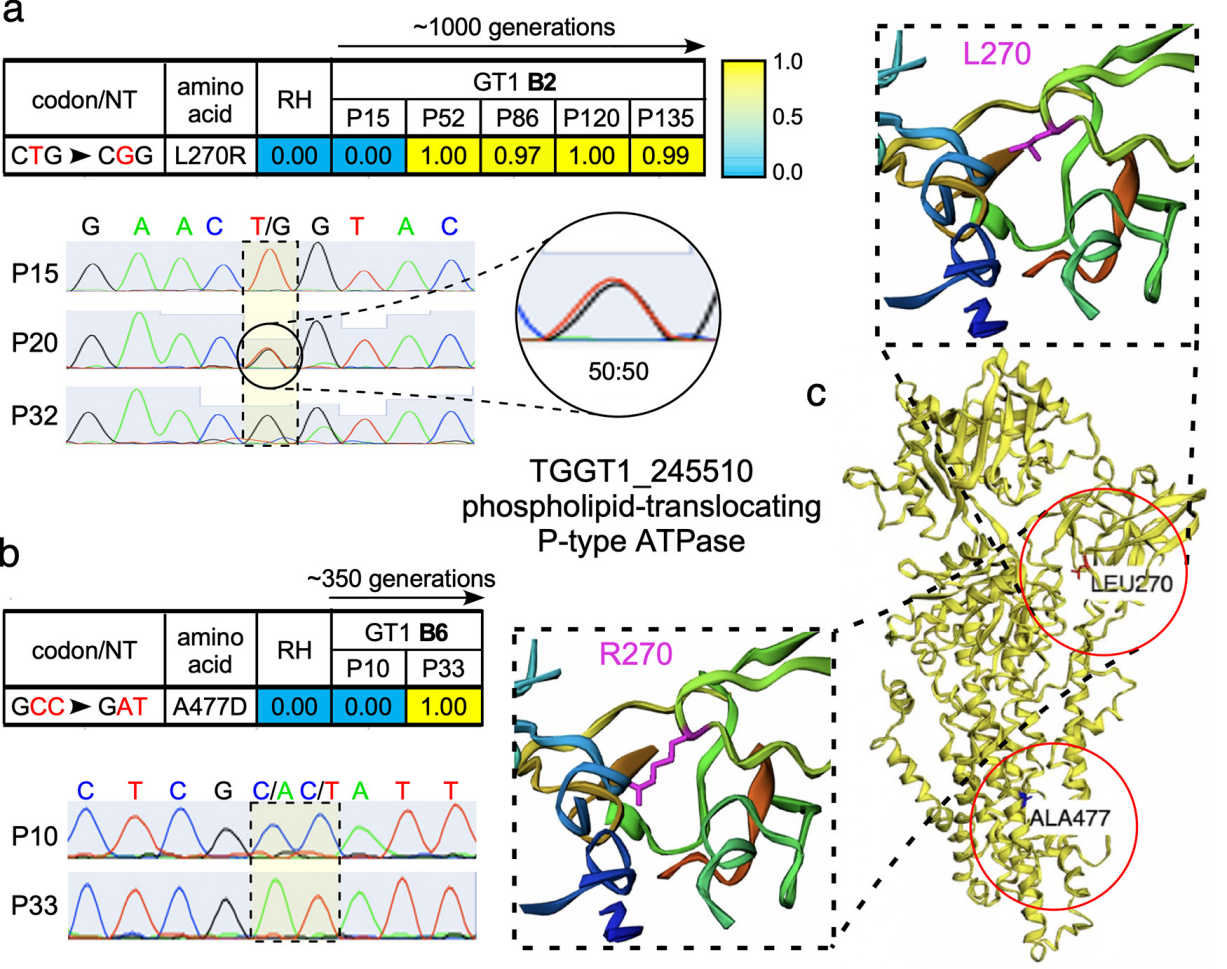
Lab adaptation identified genomic mutations within a P4-flippase gene. WGS identified the emergence of the indicated mutations with the indicated allele frequency in GT1 populations; RH is shown for reference. (a) High-frequency (≥0.75) mutations identified in GT1 population B2 or (b) GT1 population B6. (Top) Allele frequency represents the percentage of reads with the indicated allele. (Lower) Chromatogram of Sanger-sequenced PCR products confirms the presence of the P4 flippase L270R and A477D mutations in GT1 populations B2 and B6, respectively. (c) Structural prediction of the P4 flippase protein and mapping of both mutations. Zoomed-in images of the L270 and R270 alleles reveal the difference in sidechain extension into cavity space.

In the higher B2, B4, and B6 passages, we mapped many high-quality polymorphisms across all evolution trajectories (Fig. 3 and Fig. S2). B4 was sequenced at P15, P32, P79, and P105, spanning ∼800 generations. No nonsynonymous mutation fixated in the B4 population, but two mutations in the 13th intron of a dynein heavy-chain gene were fixated by P32 (Fig. S2a). The B2 population was sequenced at P15, P52, P86, P120, and P135, spanning ∼1,000 generations (Fig. 1a). Only one nonsynonymous mutation, L270R, emerged in a phospholipid-translocating P-type ATPase (P4-flippase) gene (TGGT1_245510) and remained fixed within this evolving population (Fig. 3a). PCR plus Sanger sequencing revealed the L270R mutation as early as B2-P20 and fixating within the population by B2-P32 (Fig. 3a). The B6 population was sequenced only at P10 and P33, spanning ∼300 generations, and revealed one nonsynonymous mutation, A477D, within the same P4-flippase gene fixated by P33 (Fig. 3b). Since a nonsynonymous SNP was not detected in the RH or B4 population, there is either variation in selective pressure or routes to cope with these pressures. To gauge the genetic complexity of GT1 during lab adaptation, we sequenced five clones derived from B2-P86 (Fig. S2b). The P4 flippase L270R mutation was shared across all clones, but 16 additional mutations uniquely mapped to single clones. Although six of the mutations were nonsynonymous, none fixated in the population, suggesting that no fitness advantages were conferred by these mutations. However, the complex population structure supports the random accumulation of mutations during evolution.

10.1128/mSystems.01196-21.3 FIG S2 Mutations arise, but rarely fix, during the first 1,000 generations of *in vitro* lab adaptation. WGS identified the emergence of indicated mutations with the indicated allele frequency in GT1 populations; RH is shown for reference. (a) Mutations identified in GT1 population B4 during >800 generations of lab adaptation; 100-bp nucleotide sequence surrounding the site of mutations is shown below. (b) Mutations identified in GT1 population B2, along with five B2-P86-derived clones. N/A indicates the gene does not result in a protein product. For all panels, allele frequency represents the percentage of reads with the indicated allele. *, stop codon. Download FIG S2, TIF file, 1 MB. Copyright © 2021 Primo et al. 2021 Primo et al. https://creativecommons.org/licenses/by/4.0/ This content is distributed under the terms of the Creative Commons Attribution 4.0 International license.

The identification of two different nonsynonymous mutations in the P4 flippase gene across two parallel evolving lines strongly suggests that these changes confer critical fitness benefits during lab adaptation (13, 16). To assess potential effects on the function, we modeled the *Toxoplasma* gene and predicted the impact of the amino acid replacements on the structure using the Missense3D predictive structural analysis (17) (Fig. 3c). The L270R mutation mapped to the cytoplasmic actuator domain, which is responsible for inducing the functional conformational change by dephosphorylating the neighboring P domain (18). The mutant allele results in a 78-Å^3^ decrease in cavity space due to the longer arginine side chain. The A477D mutation is within the α-helix of the ATPase transmembrane domain. It is therefore conceivable that these mutations affect the efficiency and/or localization of the flippase, although further functional analysis is required.

To assure ourselves that the low incidence of genomic mutations fixating in the population was not due to a low mutation rate, we evaluated the genomic mutation rate throughout our experiment. The clones sequenced 71 passages apart (B2-P15 and five clones at B2-P86; Fig. S2b) were used to determine the mutation rate of GT1 at 1.1 × 10^−10^ mutations/bp/generation (Fig. S3). This is within the range of RH’s reported mutation rate, 5.8 × 10^−11^ mutations/bp/generation (19), and predicts that 2 to 4% of a population accumulates a single mutation within a single passage. Thus, the lack of mutations fixating in the population is not due to an underpowered experimental design.

10.1128/mSystems.01196-21.4 FIG S3 Estimating the mutation rate in the T. gondii GT1 strain. (a) Plaque assays and cell counts before and after serial passaging were used to determine the number of viable cells and generations/passages, respectively. The number of generations T. gondii will undergo per passage correlates with the number of viable cells that are passed, which is also dependent on how lab adapted the population is. (b) Published mutation rates for Drosophila melanogaster (88), Plasmodium falciparum (89), Escherichia coli (11), Schizosaccharomyces pombe (90), Saccharomyces cerevisiae (91), T. gondii RH strain (19), and our calculated mutation rates for the GT1 subclones isolated for B2-P86. Download FIG S3, TIF file, 0.2 MB. Copyright © 2021 Primo et al. 2021 Primo et al. https://creativecommons.org/licenses/by/4.0/ This content is distributed under the terms of the Creative Commons Attribution 4.0 International license.

### Lab adaptation of GT1 results in few transcriptomic changes in the intracellular parasite.

**FIG 4:**
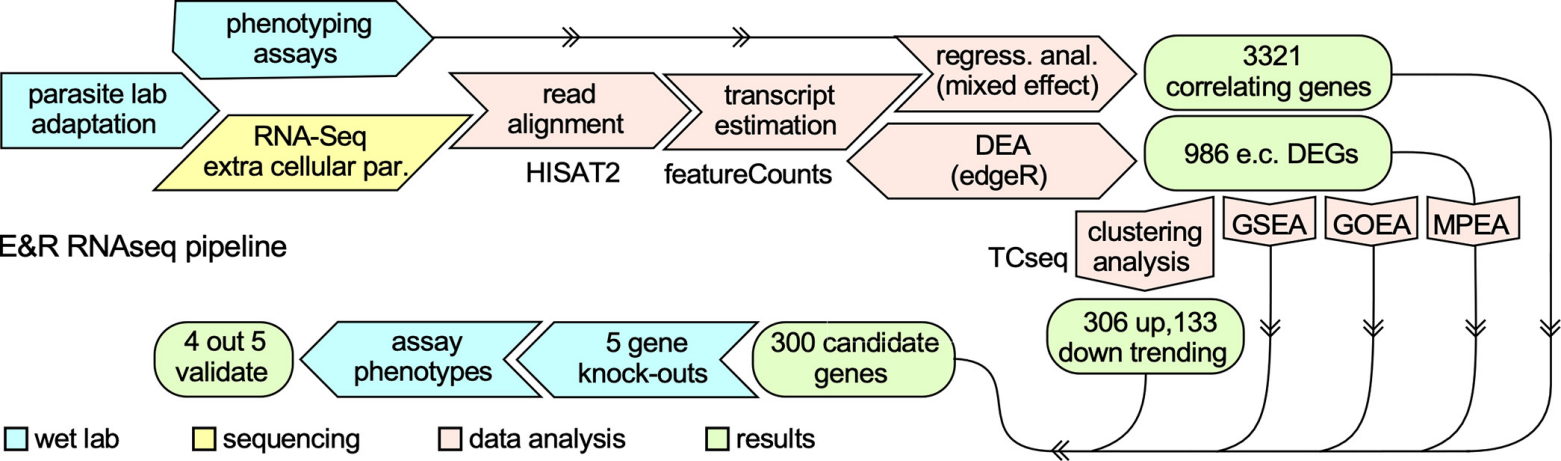
RNA-Seq analysis pipeline. Following lab adaptation, 6-h extracellular GT1 parasites at various time points were subjected to short-read mRNA sequencing (Illumina). Read alignment with HISAT2 and transcript estimation with featureCount preceded regression analysis (RA) using a mixed-effect model and differential expression analysis (DEA) with edgeR. RA identified 3,321 phenotype-correlating genes. DEA identified 988 differentially expressed genes (DEGs) relative to the earliest passage GT1 clone (B2-P11). Clustering the detected DEGs with time course sequencing (TCseq) identified up- and down-trending gene clusters. The overlap of genes identified by DEA, TCseq, and RA identified 300 genes to be differentially expressed, correlating with at least one of the phenotypes, and trending up or down with the passage of time, thereby serving as candidate *in vitro* virulence factors that will likely provide additional insights into GT1’s extracellular adaptation. Gene set, gene ontology, and metabolic pathway enrichment analysis (GSEA, GOEA, and MPEA, respectively), along with life stage analysis, characterized the 986 DEGs and provided biological insights. Five representative upregulated genes (with a neutral fitness effect on the lytic cycle; score between +1.0 and −2.5 [28]) were selected for validation of their phenotype-conferring capacity by generating knockouts in a high-passage-number B2 strain.

The single genomic mutation fixating in the population does not track with the continuing increase in plaque size during GT1’s lab adaptation. We reasoned transcriptomic changes might be the mechanism controlling lab adaptation traits. To this end, we performed mRNA sequencing (RNA-Seq) on asynchronously replicating intracellular GT1 B2 parasites at passages P11, P84, and P148. Read alignment and transcript estimation with HISAT2 (20) and featureCounts (21), respectively, were applied to all sequenced samples to obtain the read counts that were then used for downstream analysis (Fig. 4). Differential expression analysis (DEA) of P84 and P148 relative to the earliest time point, B2-P11, only identified 12 DEGs with 9 genes annotated as hypothetical (Table S1, Tab 1). The limited number of DEGs indicated that the intracellular state of GT1’s lytic cycle is not affected by lab adaptation, corroborating the phenotypic analysis of intracellular virulence traits.

10.1128/mSystems.01196-21.1 TABLE S1 Tab 1, 12 DEGs identified in intracellular GT1 parasites during lab-adaptation. RNA-seq of intracellular B2 GT1 populations at passages P11, P84, P148, followed by DEA, identified 12 significant DEGs (FC ≥ 2, *q*  ≤ 0.05) relative to the lowest passage, P11. Log_2_ fold change is show; red text indicates statistical significance (*q* ≤ 0.05). Tab 2, 986 DEGs identified in 6-h extracellular GT1 parasites during lab adaptation. RNA-seq of 6-hr extracellular B2 GT1 populations at passages P11, P35, P55, P85, P148, and P210, followed by DEA, identified 986 significant DEGs (FC ≥ 2, *q*  ≤ 0.05), relative to the lowest passage, P11. Of those, 435 DEGs were upregulated and 551 DEGs were downregulated. Log_2_ fold change is shown; red text indicates statistical significance (*q* ≤ 0.05). Tab 3, GSEA, GOEA, and MPEA of the 986 DEGs and 300 DEGs identified in 6-h extracellular GT1 parasites during lab-adaptation. GSEA, GOEA, and MPEA was performed on upregulated and downregulated DEG identified in Tab 2 (986 DEGs) and Tab 5 (300 DEGs). GOEA and MPEA results were obtained from ToxoDB.org. Tab 4, free fatty acid analysis of RH and GT1-B2 passages P11 and P206, including the C_14:0_ mystic acid and C_16:0_ palmitic acid standards. Highlighted rows mark the peak signals used for further analyses and calculations. Medium-chain FAs are defined as C_14:0_ and C_16:0_; long-chain FAs are defined as C_18:0_, C_18:1_, and C_19:0_. Statistical analysis is included. Tab 5, 300 DEGs identified in 6-h extracellular GT1 parasites during lab adaptation. Refined list (derived from Tab 2) of the highest phenotype correlating (R^2^ ≥ 0.70, Spearman correlation) DEGs, as identified by the overlap of DEGs identified by DEA, TC-seq, and RS analysis. Of the 300 DEGs, TC-seq identified 3 main expression trends (i.e., clusters) identified as clusters 1, 2, and 6. Cluster 1 consists of 119 upregulated DEGs; cluster 2 consists of 107 downregulated DEGs; and cluster 6 consists of 74 upregulated DEGs. The Spearman correlation coefficient of all 300 DEGs is provided. Tab 6, 300 DEG subcellular localizations. HyperLOPIT subcellular localization data available for tachyzoites (27) were downloaded for ToxoDB and, in case of discrepancy between the statistical methods, manually curated where possible, including assignment of two transcription factors to the nuclear pool. The mixed pool refers to where a call could not be made on the conflicting statistical assignments. Tab 7, expression patterns of epigenetic markers and transcription factors in extracellular parasites over the evolutionary time. Differential expression was determined using FC ≥ 2, *q*  ≤ 0.05 cutoffs. Factors were considered to be differentially expressed if in at least one of the passages relative to initial time point P11 the FC and *q* value cutoff values were met. The goodness of fit (*R*^2^) as well as the slope and the intercept are indicated in the table. Genes with *R*^2^ ≥ 0.5 were considered trending (up if the slope is positive and down if the slope is negative). Tab 8, 2,393 DEGs identified between intracellular and 6-h extracellular GT1 parasites. RNA-seq of intracellular and 6-h extracellular RH and GT1 (B0-P7) parasites, followed by DEA, identified 2,393 DEGs that are significantly (FC ≥ 2, *q*  ≤ 0.05) different between the intracellular and extracellular state in both RH and GT1 (B0-P7) parasites. Of those, 1,189 DEGs are upregulated and 1,204 DEGs are downregulated. Other GT1 passages are also shown for comparison. Log_2_ fold change is shown; red text indicates statistical significance (*q* ≤ 0.05). Tab 9, oligonucleotide sequences used. Table provided indicates oligonucleotide sequence used for M13 primers used for PCR-Sanger sequencing of known RH and GT1 SNPs and for genotyping the P4-flippase (TGGT1_2455510) SNPs; protospacer sequences used to generate KOs; primers used to generate DHFR cassette used as a repair template; diagnostic PCR primers used to confirm genomic integration of DHFR cassette; and qRT-PCR primers used to confirm mRNA ablation in KOs. Download Table S1, XLSX file, 0.5 MB. Copyright © 2021 Primo et al. 2021 Primo et al. https://creativecommons.org/licenses/by/4.0/ This content is distributed under the terms of the Creative Commons Attribution 4.0 International license.

**FIG 5:**
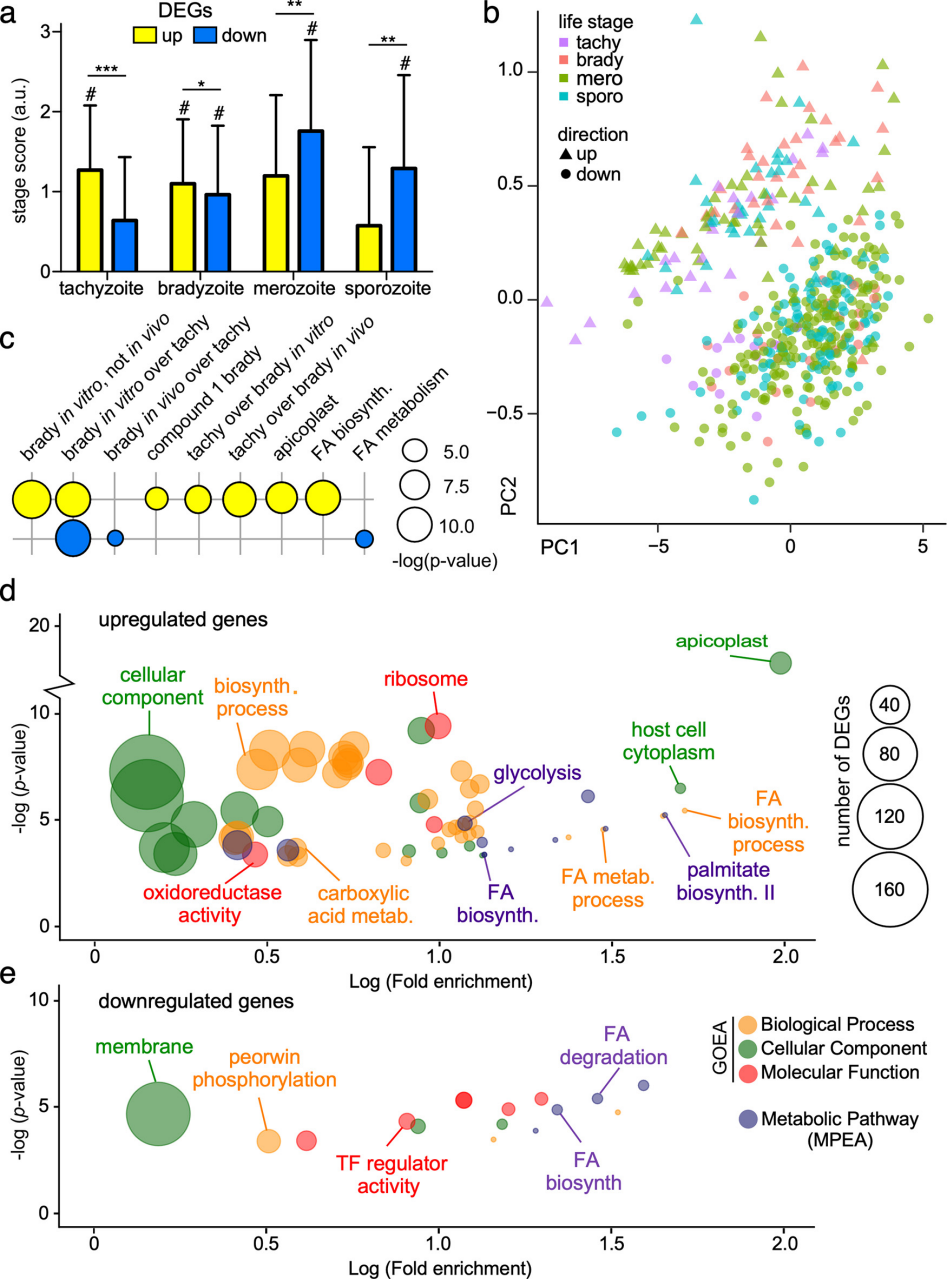
Biological insights of GT1’s evolved extracellular transcriptome. (a) Life stage analysis was utilized to characterize the types of life stage genes that are differentially expressed during GT1’s lab adaptation; #, *P* ≤ 0.05, as determined by an independent bootstrap analysis (*n* = 1,000 random sampling) for each individual life stage; *, *P* < 10^−8^; **, *P* < 10^−16^; ***, *P* < 10^−32^, as determined by two-tailed *t* test. (b) PCA plot of changes in the expression of 469 genes from the 989 differentially expressed genes during GT1’s lab adaptation that were uniquely representing a single life stage, as indicated (36 tachyzoite-, 53 bradyzoite-, 240 merozoite-, and 140 sporozoite-representing genes). (c) Gene set enrichment analysis (GSEA) (c) as well as GOEA (d) and MPEA (e) of up- and downregulated DEGs identified an enrichment in FA metabolism genes being largely upregulated in the apicoplast.

10.1128/mSystems.01196-21.1 TABLE S2 Source data. Tab 1, Fig. 1c. Tab 2, Fig. 1d. Tab 3, Fig. 2a. Tab 4, Fig. 2b. Tab 5, Fig. 2c. Tab 6, Fig. 2d. Tab 7, Fig. 5a. Tab 8, Fig. 5, 6 log CPM. Tab 9, Fig. 5, 6 q values. Tab 10, Fig. 5c, phenotype Spearman correlations. Tab 11, Fig. 7e. Tab 12, Fig. 7f. Tab 13, Fig. S1a, b. Tab 14, Fig. S1c. Tab 15, Fig. S1d. Tab 16, Fig. S3a. Tab 17, Fig. S3b. Tab 18, Fig. S5. Tab 19, Fig. S7c. Download Table S2, XLSX file, 16 MB. Copyright © 2021 Primo et al. 2021 Primo et al. https://creativecommons.org/licenses/by/4.0/ This content is distributed under the terms of the Creative Commons Attribution 4.0 International license.

### Lab adaptation of GT1 is associated with many pro-tachyzoite, transcriptomic changes in extracellular parasites.

Given the prominent phenotype adaptations in extracellular parasites, we hypothesized that differential gene expression might be associated with this state. RNA-Seq, transcript abundance estimation, and DEA on 6-h extracellular B2 GT1 populations at passages P11, P35, P55, P85, P148, and P210 identified 986 significant DEGs (fold change [FC] ≥ 2, *q* ≤ 0.05) relative to the earliest passage, P11 (Fig. 4). Of those, 435 DEGs were upregulated and 551 DEGs were downregulated (Table S1, Tab 2). Nearly 55% of these DEGs are of unknown function, challenging our ability to interpret the biology of the entire data set. Regardless of gene annotations, previously published RNA-Seq data sets allowed us to characterize these DEGs in the context of T. gondii*’s* four life cycle stages: tachyzoite, bradyzoite, merozoite, and sporozoite (22–24). We first developed a scoring method to assess how such genes are differentially expressed across all four developmental life stages (Fig. S4a). We validated this method by scoring previously published tachyzoite-, bradyzoite-, and sporozoite-associated gene sets (25). Indeed, each gene set only scored significantly (*P* ≤ 0.05) for their respective life stage, validating our scoring approach (Fig. S4b to d). Application of this scoring scheme to the evolved DEG data set revealed a significant upregulation of tachyzoite-associated genes and significant downregulation of merozoite- and sporozoite-associated genes (Fig. 5a). In parallel, we also defined gene sets comprising genes uniquely expressed in each life stage. Overall, 469 out of 986 genes could be uniquely assigned to a single life stage. The scaled (z-score) log counts per million (CPM) expression values of the 469 genes were used to generate a heatmap (Fig. S4e, left). Genes in each life stage were ordered using hierarchical clustering with Euclidean distance. A similar heatmap was generated using log_2_ FC values relative to the initial time point, P11 (Fig. S4e, right). Tachyzoite-specific genes were mostly upregulated, whereas merozoite and sporozoite genes were mostly downregulated. The bradyzoite-associated gene repertoire was almost equally up- and downregulated, presumably due to the stress response to the extracellular condition that may be (partly) shared with bradyzoite differentiation. Mean log CPM expression values across the three biological replicates were used to perform principal component analysis (PCA). PCA projection shows a separation between up- and downregulated genes (Fig. 5b). Overall, these analyses indicate that in extracellular parasites, lab adaptation leads to a reduction in merozoite and sporozoite gene expression and upregulation of tachyzoite genes, resulting in a more protachyzoite profile.

10.1128/mSystems.01196-21.5 FIG S4 Developing and validating T. gondii*’s* life stage score analysis. (a) Available tachyzoite, bradyzoite, merozoite, and sporozoite RNA-seq data sets (ToxoDB.org; see Materials and Methods) (22–24) were utilized for differential expression analysis (DEA). Each stage was compared to the three other stages (e.g., tachyzoite versus bradyzoite; tachyzoite versus sporozoite; tachyzoite versus merozoite). The number of times each gene was significantly upregulated in this 3-way DEA was enumerated to yield a maximum score of 3 or minimum score of 0 for each gene. The average enumeration of previously published lists (25) of genes was calculated for tachyzoite (b)-, bradyzoite (c)-, and sporozoite (d)-associated genes. Color-coded hashtags (#) indicate *P* value of ≤0.05, as determined by an independent bootstrap analysis (*n* = 1,000 random sampling) for each life stage. Error bars indicated SD. (e) Heatmaps of the normalized expression of genes uniquely expressed in the life stage as indicated. Unique life stages genes were defined by a 3-way stage score of 3. Left, normalized expression; right, normalized log2 fold change value of each time point relative to P11. Download FIG S4, TIF file, 1.2 MB. Copyright © 2021 Primo et al. 2021 Primo et al. https://creativecommons.org/licenses/by/4.0/ This content is distributed under the terms of the Creative Commons Attribution 4.0 International license.

### Gene enrichment analyses suggest fatty acid biosynthesis as a lab-adaptive biological process in the extracellular milieu of GT1’s lytic cycle.

**FIG 6:**
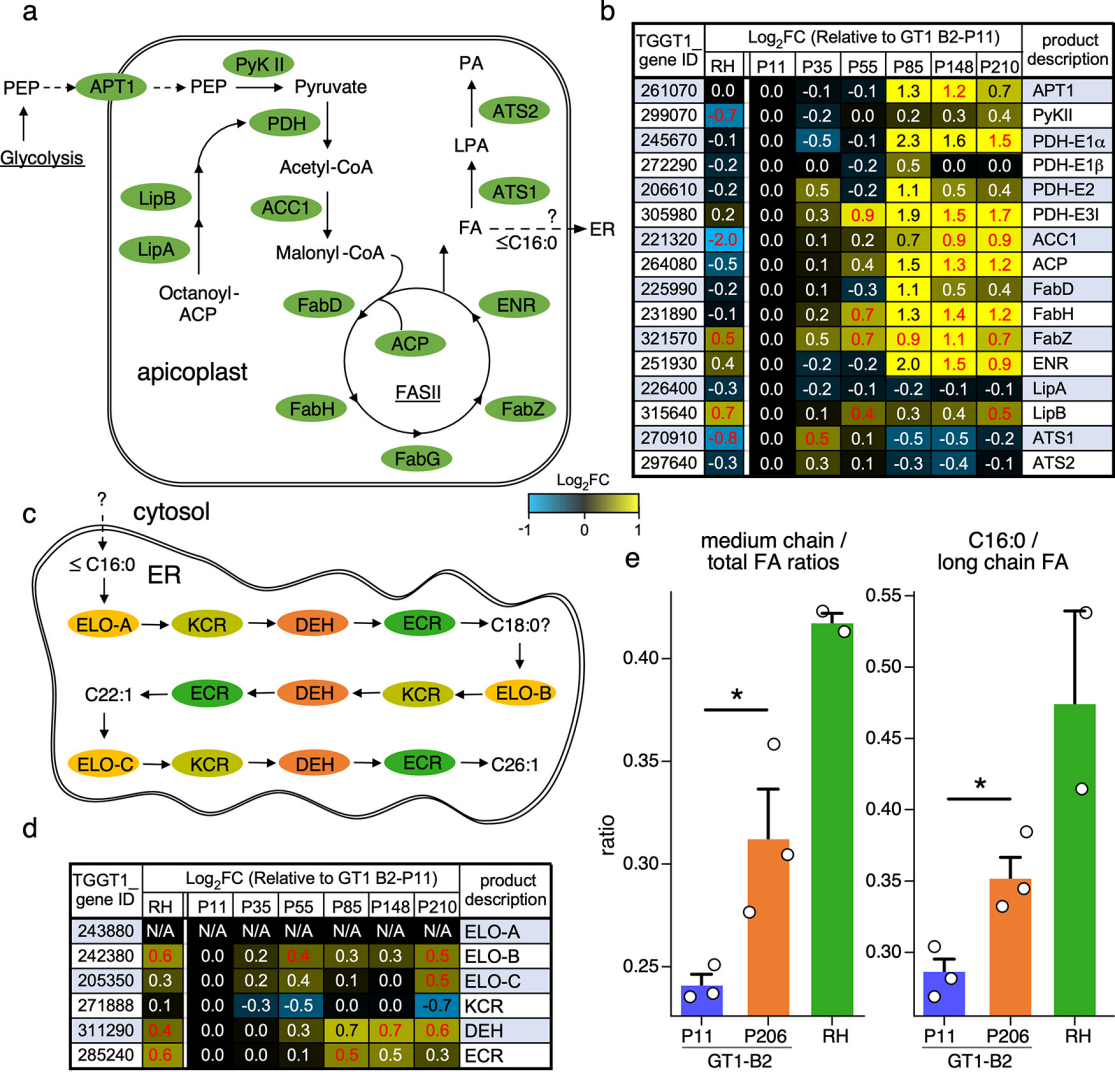
FASII pathway becomes upregulated in extracellular GT1 during lab adaptation. (a) Glycolysis produces PEP, which is transported into the apicoplast by APT1 and converted into pyruvate by PyKII (84). Once lipoylated by LipA/B, the PDH complex converts pyruvate into acetyl-CoA, which is then metabolized to generate malonyl-CoA, the precursor metabolite required for the FASII pathway and FA synthesis (85, 86). (b) Expression of the genes involved in the multistep process of *de novo* fatty acid synthesis within the apicoplast. (c) Medium-chain FA are translocated from the apicoplast to the ER for repeated rounds of carbon chain elongation by ELO A/B/C, KCR, DEH, and ECR (87). (d) Expression of several genes involved in FA elongation within the ER. Red text indicates a *P* value of ≤0.05. (e) Analysis of free FA species ratios in parasite strains as indicated, expressed in two different ways. GT1 B2-P11 parasites have a significantly lower abundance of medium-chain (C_14:0_, C_16:0_) FAs than high-passage-number (P206) GT1-B2 parasites or the RH strain. Average ± standard errors of the means is plotted; *n* = 3 (*n* = 2 for RH). *, *P* < 0.05.

To distill biological insights from the 986 DEGs associated with lab adaptation, we employed three types of statistical enrichment analyses (Fig. 4): gene set enrichment analysis (GSEA [25]), gene ontology enrichment analysis (GOEA), and metabolic pathway enrichment analysis (MPEA) (Table S1, Tab 3). The upregulation of genes in the apicoplast gene set recurred in GSEA and GOEA with statistical significance (adjusted *P* value [adj-*P*] < 0.05). The upregulation of FA was identified across all three analyses with a *P* value of <0.05; however, upon multiple testing correction, the adj-*P* value only remained significant for upregulation of FA biosynthesis in the GSEA (adj-*P* = 0.006). Both MPEA and GSEA show significant upregulation of FA biosynthesis and FA metabolism, although neither term remains significant after multiple testing correction (Fig. 5c to e). Specifically, the majority of genes in the apicoplast’s FASII pathway became upregulated during GT1’s lab adaptation (Fig. 6a and b). While medium-length carbon chain FAs produced in the apicoplast can be further elongated in the endoplasmic reticulum (ER), we only observed a modest upregulation of the FA elongation pathway (Fig. 6c and d). To validate if these transcriptional profiles resulted in actual changes at the FA level, we analyzed the free FA composition in GT1-B2 at the beginning and end of the evolutionary trajectory. Indeed, medium-chain FAs (C_14:0_ and C_16:0_) were underrepresented at P11 relative to the high passage numbers and RH (Fig. 6e and Table S1, Tab 4). Taken together, these data strongly suggest that FA availability is a selective pressure in the extracellular milieu.

### Acquired *in vitro* virulence is a polygenic trait sustained by gene expression.

**FIG 7:**
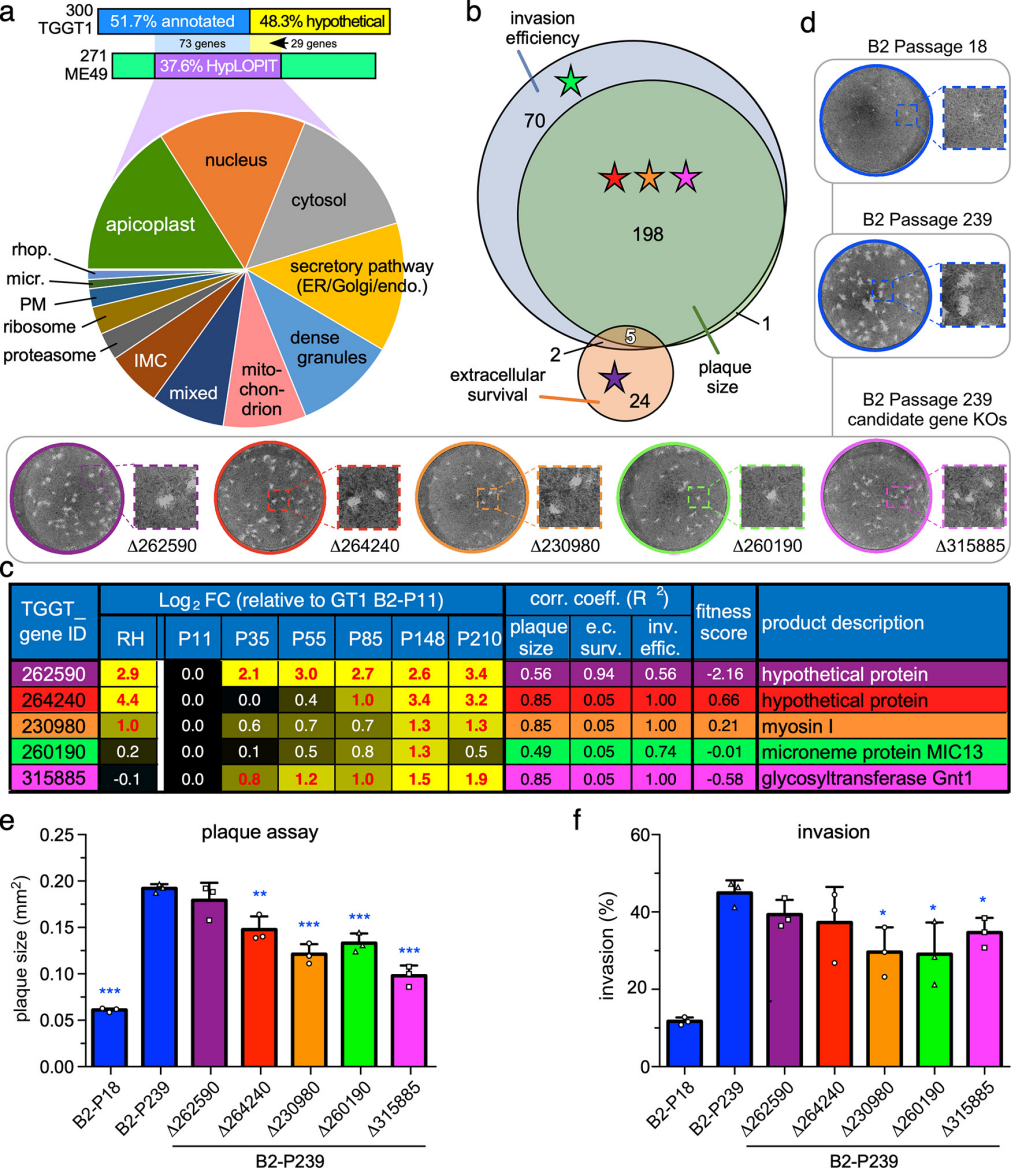
Functional analysis of candidate gene knockouts identified several differentially expressed genes important for optimal *in vitro* virulence, suggesting acquired *in vitro* virulence is a polygenic trait. (a) HyperLOPIT subcellular localization data (27) on the 300 strongest phenotype-correlating genes. Only 271/300 genes are cross annotated between the TGGT1 and TGME49 genome annotation (data are only available for the ME49 genes). Ninety-seven of the 107 genes with HyperLOPIT data are upregulated. The mixed slice comprises genes with conflicting assignments between the two statistical algorithms used for the subcellular assignments. Data are available in Table S1. GOEA and GMEA for this gene set are provided in Fig. S6. (b) Venn diagram of the 300 strongest phenotype-conferring genes and their correlation with lab-adaptive phenotypes. (c) Expression profiles (log_2_ FC), phenotype correlation coefficients (*R*^2^), and fitness score in the genome-wide CRISPR screen of the lytic cycle (28) of the five genes chosen for KO. Red text indicates *P* ≤ 0.05. (d to f) Upon successful KO (Fig. S7), plaquing capacity after 11 days (e) and invasion capacity (f) were evaluated. Mean of ≥3 biological replicates (represented as squares, triangles, or circles) with SD is plotted. Blue asterisks indicate the *P* value of the indicated GT1 passages relative to B2-P239; *, *P* ≤ 0.05; **, *P* ≤ 0.01; ***, *P* ≤ 0.001. For all panels, the five genetic KOs are color coded. iKD, inducible knockdown.

We performed several analyses to calculate gene trends and correlation of genes with phenotypes to further refine the list of 986 DEGs (Fig. 4). The steps taken are as follows. (i) A linear mixed-effect regression model with smoothing B-splines was used to fit the time course gene expression and phenotype data. (ii) The inferred mean curves were sampled at regular intervals to align the phenotype and gene expression data and to calculate the correlation between each phenotype and gene expressions. (iii) Genes whose expression strongly correlated (*R*^2^ ≥ 0.70, Spearman correlation) with GT1’s evolved phenotypes were identified. (iv) A time course clustering algorithm (TCseq) was employed to identify groups of genes with similar patterns of expression over time. A regression line was then fitted to the genes in each cluster to quantify the overall trend (26) (Fig. S5). (v) Finally, the overlap of 986 DEGs that correlated highly with a phenotype (3,321 total) and trending genes (306 trending up, 138 trending down) was calculated, resulting in a final list of 300 trending and phenotype-correlating genes (Table S1, Tab 5). The unbiased tachyzoite HyperLOPIT proteome analysis (27) provided the subcellular localization for 131 of these gene products (Fig. 7a and Table S1, Tab 5). This consolidated the prominent role for the apicoplast and highlights the secretory pathway as potential pressure for needs of lipids. Interestingly, the data covered 122 of the 193 upregulated genes but only 9 of the 107 downregulated genes, which makes the latter set rather enigmatic (this set was also sparse in GOEA and MPEA) (Fig. S6 and Table S1, Tab 5). Correlation of the 300 genes with the trends in phenotype evolution linked 31 genes with extracellular survival, 204 genes with plaque size, and 275 genes largely overlapping with plaque size, correlated with invasion efficiency (Fig. 7b). To obtain experimental support for the phenotype-correlating genes as potential *in vitro* virulence factors, we selected five genes spanning the phenotypic associations for genetic disruption (Fig. 7b, colored stars). Exclusively up-trending genes were selected, which granted us the ability to knock out (KO) genes in high-passage-number GT1 B2-P239 with minimal effects of lab adaptation during the time it took to isolate the KO mutant. In addition, to avoid KO of essential genes, we focused on genes with a neutral fitness-conferring effect (i.e., fitness score between +1.0 and −2.5), as identified in the genome-wide CRISPR screen (Fig. 7c) (28). We selected a glycosyltransferase (Gnt1; an E3-ubiquitin ligase [29]), motor protein myosin I (MyoI; facilitates cell-cell communication during division [30]), microneme protein 13 (MIC13; has been associated with oxidative stress survival through an unknown mechanism [31]), and two hypothetical genes (TGGT1_262590 and TGGT1_264240) (Fig. 7c). Genotypes of isolated clones were confirmed by diagnostic PCR and the ablation of mRNA expression by reverse transcription-PCR (RT-PCR) (Fig. S7). The five KO clones were evaluated for plaque size and invasion efficiency using low-passage-number B2-P18 and the B2-P239 parent as controls. Relative to B2-P239, four KO lines showed a reduction in plaque size (0.51- to 0.76-fold; *P* < 0.01) (Fig. 7d and e), while three lines displayed a reduced invasion efficiency (0.65- to 0.77-fold; *P* < 0.05) (Fig. 7f). Importantly, the KO parasites displayed the phenotypic characteristics predicted by the regression model (*R*^2^) (Fig. 7c), indicating a high degree of accuracy in phenotype prediction from our mixed-effect regression splines. Upregulation of MyoI and Gnt1 strongly correlated with plaque size and invasion efficiency, which was directly validated in the KO phenotypes of these genes. On the other hand, TGGT1_262590 did not show strong correlation with plaque size and invasion efficiency during lab adaptation, and its KO validated that relationship. Based on each gene’s plaque size and invasion correlation coefficient, only two unexpected outcomes were observed: TGGT1_264240-KO displayed reduced invasion efficiency of ∼17%, as expected by RA (*R*^2^ = 1.0), but this did not reach statistical significance (*P* = 0.24) due to the sizable standard deviation in our biological replicates (Fig. 7f); MIC13-KO displayed a significant ∼35% reduction in plaque size, which was much more dramatic than expected given its *R*^2^ value of only 0.49. Overall, the predicted phenotypes for four out five genes were successfully confirmed. This strongly suggests that the list of 300 phenotype-correlating, trending genes (Table S1, Tab 5) truly harbors many *in vitro* virulence factors. This indicates that GT1’s acquired *in vitro* virulence is a polygenic trait sustained by specific gene expression patterns.

10.1128/mSystems.01196-21.6 FIG S5 Up- and down-trending gene clusters identified by TCseq. (a) Normalized expression of all identified gene clusters. The genes comprising the 300 candidate DEGs identified by DEA, TCseq, and RA are contained in clusters 1, 2, and 6, since only these 3 show the most linear up or down trends across all time points, consistent with the steady developments in phenotypes. (b) Heatmaps of the 439 genes genes in clusters 1, 2, and 6. This gene set was intersected with the phenotype-conferring score and resulted in the selection. Download FIG S5, TIF file, 1.9 MB. Copyright © 2021 Primo et al. 2021 Primo et al. https://creativecommons.org/licenses/by/4.0/ This content is distributed under the terms of the Creative Commons Attribution 4.0 International license.

10.1128/mSystems.01196-21.7 FIG S6 GOEA for the 300 most trending genes. Top, up trending; bottom, down trending. Download FIG S6, TIF file, 0.3 MB. Copyright © 2021 Primo et al. 2021 Primo et al. https://creativecommons.org/licenses/by/4.0/ This content is distributed under the terms of the Creative Commons Attribution 4.0 International license.

10.1128/mSystems.01196-21.8 FIG S7 Generating genetic knockouts of regression analysis candidates by CRISPR/Cas9. (a) Strategy for generating KO of candidate DEGs. Transfection of one or two CRISPR plasmids generates a double-strand break, allowing for recombination of a cotransfected DHFR selection cassette with short homologous flanks, into the genetic locus and disrupting the gene of interest. Note that sites of recombination are not drawn to scale; relative regions for diagnostic PCR to confirm integration and qRT-PCR to confirm ablation of mRNA expression are shown. (b) Diagnostic PCR of clonal KO parasites confirms integration of the DHFR cassette into the gene of interest. (c) qRT-PCR of clonal KO parasites confirms ablation of mRNA expression of the gene of interest. Fold change, relative to the parental line (B2-P239) with SD, is shown; N.D. indicates the expression was not detected. Download FIG S7, TIF file, 0.4 MB. Copyright © 2021 Primo et al. 2021 Primo et al. https://creativecommons.org/licenses/by/4.0/ This content is distributed under the terms of the Creative Commons Attribution 4.0 International license.

### The epigenetic and transcriptional networks of lab adaptation and the extracellular state.

**FIG 8:**
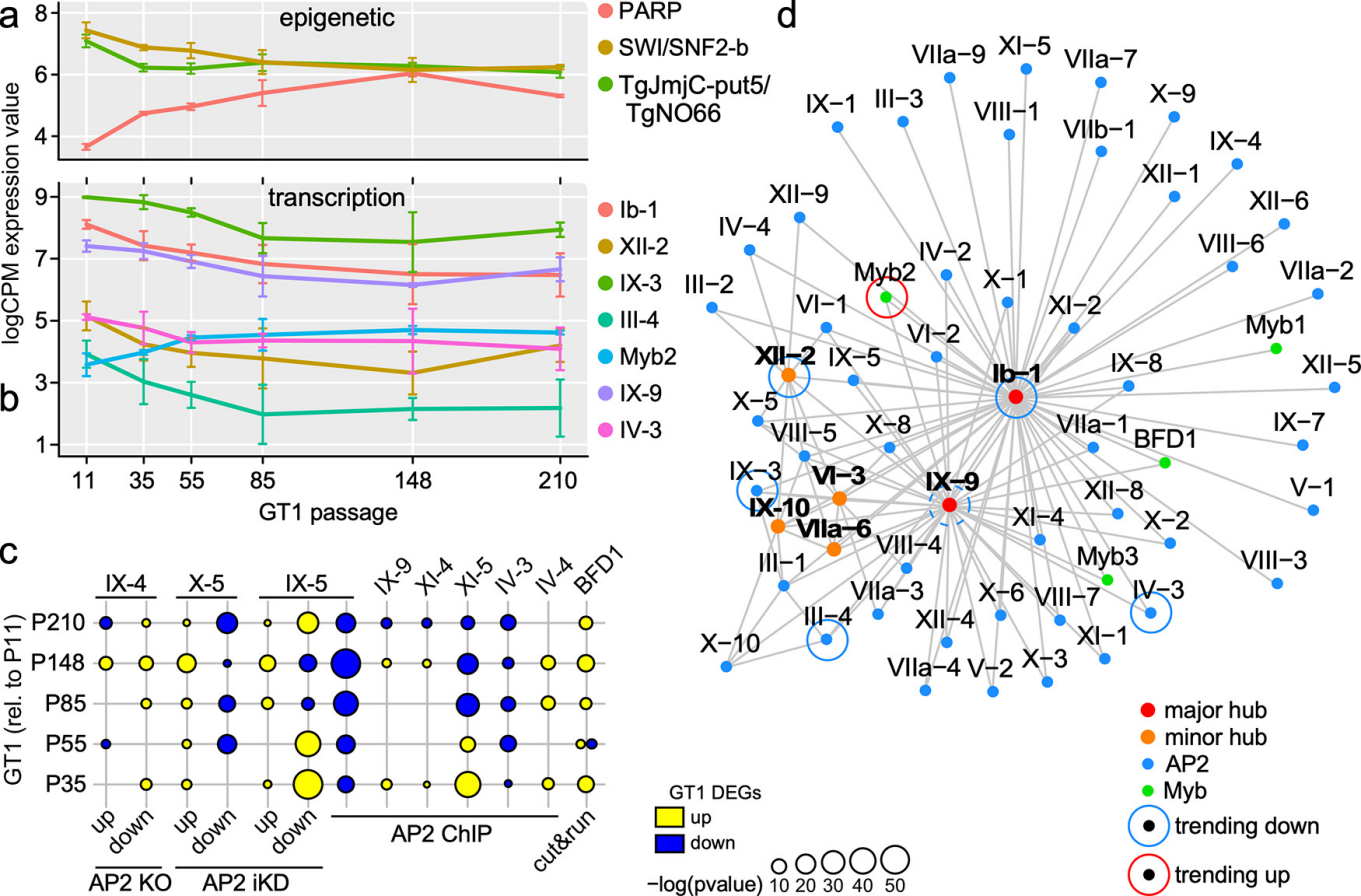
Gene expression network analysis with AP2 and Myb family transcription factors. (a) Inspecting the expression of epigenetic factors in extracellular parasites identified two down-trending and one up-trending factor representing a statistically significant trend over time based on *R*^2^ (≥0.5) of the regressed line to each of the epigenetic factor’s expression level (Table S1, Tab 7). (b) Inspecting the expression of AP2 and Myb TFs in extracellular parasites identified six down-trending AP2s and one up-trending Myb among which AP2Ib-1, AP2IX-3, AP2IV-3, and Myb2, representing a statistically significant trend over time based on *R*^2^ (≥0.5) of the regressed line to each of the TF’s expression level (Table S1, Tab 7). Although AP2IX-9 does not reach the statistical significance level of trending, its expression level is significantly changing between P11 and P148. Trends of the AP2s indicate their key role in the extracellular transcriptional mechanism. (c) Previous studies have identified direct (by AP2 ChIP-Seq [14], ChIP-qPCR [38, 40, 42], ChIP-chip [43] or cut and run [44]) and indirect (by AP2 ikD or KO followed by RNA-seq [14, 15] or microarray analysis [41]) targets of AP2 and Myb TFs. GSEA comparing these direct/indirect transcription factor targets to lab-adaptive DEGs identified significant enrichments, indicating AP2 TFs as potential regulators of lab adaptation. (d) Transcription factor expression network analysis incorporating both intracellular and extracellular RNA-Seq data over the evolutionary trajectory based on predicted AP2 and Myb transcription factors. The following Myb domain genes were considered: TGGT1_200385 (BDF1), TGGT1_203950, TGGT1_213890, TGGT1_264120 (Myb1), TGGT1_275480, TGGT1_306320 (Myb2), TGGT1_321450 (Myb3), and TGGT1_215895. AP2IX-10 represents TGGT1_215895, which was not previously named.

We sought to define the epigenetic and transcription factors that orchestrate the transcriptomic changes during lab adaptation. Posttranslational histone modifications have been associated with tachyzoite cell cycle progression as well as differentiation between the T. gondii life stages (32, 33). Here, we assembled a list comprising 96 validated and putative epigenetic factors encoded by the T. gondii genome (33–37) and determined if their expression levels changed over the course of lab adaptation (Table S1, Tab 7). By comparing expression levels at B2-P210 with B2-P11, we identified three factors in extracellular parasites, none of which have been experimentally studied, TgPARP (TGGT1_270840), which is trending up, and two that trended down, SWI/SNF2-containing protein-b (TGGT1_277070) and the putative histone demethylase TgJmjC-put5/TgNO66 (TGGT1_240840) (Fig. 8a). Further work is needed to determine the function of these factors and their specific roles in lab adaptation.

At the transcriptional level, the apicomplexan Apetala 2 (ApiAP2) family of transcription factors (TFs) spans 68 annotated members and have been associated with numerous traits (14, 15, 38–43). In addition, Myb TFs are another significant family (14 annotated genes on ToxoDB) associated with specific functions. We identified seven TFs whose expression in extracellular parasites significantly changed during lab adaptation (Fig. 8b and Table S1, Tab 7; note that AP2IX-9 is significant at P85 and P148 but not at P210). All six AP2 factors are trending down, while a Myb TF, which we named Myb2 (TGGT1_306320), trends up. Interestingly, three down-trending AP2s, Ib-1, IV-3, and IX-9, have specific roles in bradyzoite differentiation: they are upregulated at the start of differentiation but are downregulated in mature bradyzoites (38). The targets of AP2IV-3 and AP2IX-9 have also been associated with cyst formation. AP2IX-9 expression peaks early in tissue cyst formation and actually suppresses it, while AP2IV-3 promotes this process and peaks later (38). We intersected the identified targets of these factors (and all other AP2s for which target data are available) with the list of DEGs during lab adaptation (Fig. 8c) (38). Indeed, AP2IV-3-controlled genes trend down, but the effect is much weaker for AP2IX-9. Therefore, lab adaptation seems to be driven beyond the inhibition of state change controlled by APIX-9. To be comprehensive, we repeated this analysis for all TFs with known targets (14, 15, 38, 40–44). Genes controlled by BFD1, the master regulator of bradyzoite differentiation (44), are also upregulated during lab adaptation. Expression of most genes controlled by AP2XI-5 (43), AP2X-5 (15), and AP2IX-5 (14, 45) trends down, which is not surprising since these TFs are associated with cell division, and extracellular parasites do not divide (46).

To comprehensively grasp any major players of GT1’s polygenic lab adaptation, we constructed a protein-protein interaction (PPI) network of AP2s and Mybs using a Gaussian graphical model (GGM) fitted on all RNA-Seq data points across passages from both intracellular and extracellular conditions (Fig. 8d). AP2IX-9 and AP2Ib-1 appear as the main hubs in the transcriptional network. The former is a repressor, while the latter is a suspected activator of bradyzoite gene expression (38, 42, 47). Moreover, the additional connection of BFD1 to these two main hubs fits with the shared bradyzoite profile. Some of the interactions, such as the link between AP2IX-5 to AP2IX-9 and AP2XII-2, as well as the link between AP2X-5 and AP2Ib-1, were previously reported and serve as calibration points of our analysis (14, 15). Overall, our transcriptional network analysis confirms the significant overlaps with the bradyzoite differentiation process but not with the mature bradyzoites, which fits with the mixed bradyzoite profile in the life stage analysis (Fig. 5a and b). Finally, AP2IX-9 and AP2Ib-1 are the main TFs associated with the extracellular state, whereas AP2Ib-1 and, to a lesser extent, APIX-9 are key drivers of lab adaptation.

## DISCUSSION

After ∼1,500 generations of lab adaptation, GT1 displayed a significant evolution of its *in vitro*, host-independent virulence (reproducible >2-fold plaque size increase). However, plaque size is still >2-fold smaller than that of the lab-adapted RH strain. Indeed, the steady slope of plaque size increase (Fig. 1c) indicates that at P200, GT1 is still on a continuing evolutionary path. Extracellular survival and invasion efficiency were the features most strongly evolved during lab adaptation, whereas changes in the intracellular replication cycle, as seen in RH, were not observed. Phenotype changes were driven by transcriptional reprogramming of extracellular parasites, and we identified two biological drivers, (i) a shift toward a tachyzoite-enriched gene expression profile by shutting down genes expressed in other life stages that more closely resembles RH (Fig. 5a and b) and (ii) enhanced *de novo* synthesis of FAs through upregulation of the FASII pathway (Fig. 6). Germane to the former, the RH strain has lost the ability to make mature tissue cysts as well as the ability to undergo the sexual cycle in cats, and we see this phenomenon reflected in our experiment. However, bradyzoite genes were a mix of up and down trends. Analysis of the transcriptional network revealed that several TFs associated with early-mid steps of bradyzoite differentiation (38) were upregulated during lab adaptation, which explains the upregulation of bradyzoite-associated genes. We hypothesize that the downregulated portion is primarily associated with mature bradyzoites. A key factor driving bradyzoite differentiation is stress (48), which is likely the common denominator with extracellular parasites, since these are also under environmental stress.

Pertaining to the second biological pathway, the availability of FA in the extracellular milieu seems to be a limiting factor. Further support for a critical role for lipid homeostasis is provided by the flippase gene SNPs fixating early during lab adaptation. P4-phospholipid flippases are transmembrane transporters of cations, heavy metals, and, particularly, phospholipids across lipid bilayers (49). Phospholipid flipping activity is of great biological importance for the biogenesis of vesicles (50, 51), creating a fusion-competent bilayer (52), maintaining membrane stability (53, 54), and generating signaling cues (55–57). It was recently reported that this particular *Toxoplasma* P4-flippase localizes to the endoplasmic reticulum (58), while the HyperLOPIT proteome assigned it to the Golgi-plasma membrane (27). Dissecting how the flippase SNPs modulate the kinetics or substrate preference will require extensive further work. A string of recent reports support the role of the FASII pathway in *in vitro* virulence (59–62). Furthermore, the availability of lipids in the extracellular environment has been identified as a critical factor for *in vitro* virulence in a dose-dependent manner (59, 63). The highly lab-adapted RH strain can maintain superior *in vitro* virulence even under lipid-starved conditions by upregulating its *de novo* FA/lipid synthesis by 15% (59), which supports our data (although the flippase gene in RH contains no SNPs, suggesting an alternative path to the same state). Our lab adaptation experiment was performed under 1% fetal bovine serum (FBS), which is relatively low compared to 10% FBS used by other labs and might make FAs a limiting factor. The critical insight regarding the contribution of FAs to *in vitro* virulence is the novel connection with surviving in the extracellular environment as the critical place of FA availability.

The biology of T. gondii in the extracellular milieu has been largely understudied, as it is regarded as a relatively short period bridging two intracellular replication cycles. However, ∼20% of tachyzoites in infected mouse tissue are extracellular, suggesting that tissue-residing parasites can spend significant time outside a host cell during their lytic cycle (64). Transcriptomic studies report ∼2,400 DEGs between parasites residing intracellularly versus those in the extracellular environment, a sizable portion of the 8,000+ annotated genes (Fig. S8a and Table S1, Tab 8) (24, 46, 65, 66). Since extracellular parasites are arrested in a nondividing state, we ascertained that these DEGs are not merely the result of the stalled division cycle. We intersected this gene set with genes undergoing cyclical expression throughout the intracellular replication cycle (1,967 total genes). This showed that ∼73% (1,758 genes) of the extracellular DEGs do not overlap with the cyclic transcription patterns seen in the replication cycle (Fig. S8b). Taken together, the extracellular state is transcriptionally and biologically unique but has been underappreciated for its selective pressure in lab adaptation.

10.1128/mSystems.01196-21.9 FIG S8 Extracellular milieu is a unique transcriptional state with many DEGs not related to the intracellular replication cycle. (a) Venn diagram of DEGs in our data sets identified between intracellular and extracellular RH and GT1 (B0-P7) tachyzoites. (b) Venn diagram of DEGs identified between intracellular and extracellular tachyzoites, as well as the two cycling subtranscriptomes in intracellular replicating parasites (65). Download FIG S8, TIF file, 0.2 MB. Copyright © 2021 Primo et al. 2021 Primo et al. https://creativecommons.org/licenses/by/4.0/ This content is distributed under the terms of the Creative Commons Attribution 4.0 International license.

Nearly half of the 300 genes with the highest lab adaptation correlation are hypothetical and lack protein localization data (90% of the downregulated genes fall in that class). This begs the question of their function. Insights from the experimentally validated genes hint at two processes. Upregulation of MIC13 might be part of a stress response to the extracellular environment, as indicated by a recent MIC13 study in RH growth under stressed conditions (31). Second, Gnt1 incorporates GlcNAc onto Skp1, thereby promoting the formation of the E3-ubiquitin ligase-containing SCF (Skp1/Cullin-1/F-box protein) complex (29, 67). The SCF complex directs proteins for degradation by the 26S proteasome and is O_2_ regulated in *Dictyostelium* (68), indicating its role in maintaining redox homeostasis in the cell. Interestingly, during GT1’s lab adaptation, six genes of the proteasome core complex were upregulated over time (Fig. 7a), suggesting that lab adaptation results in increased protein turnover within extracellular parasites. Lastly, the identification of MyoI is quite peculiar, as it resides in the cytoplasmic bridge of intracellular parasites and maintains parasite-to-parasite communication (30). Since extracellular parasites have no cytoplasmic bridge, MyoI might have an additional function, which we will pursue in future work. Either way, further mining of the genes associated with lab adaptation will map the nature of host-independent virulence factors and likely reveal new biological insights.

Computationally, we developed new methodologies based on mixed-effect regression with smoothing B-splines to fit the mean trends of expression and identify phenotype-correlating genes. Such approaches can be adapted for other applications involving time course gene expression data and clustering of gene curves. Moreover, we presented a novel application of regularized GGMs that identified the interaction map between AP2 and Myb genes in extracellular parasites. This model can be extended to more generally identify gene regulatory networks in T. gondii.

In conclusion, our work demonstrates that lab adaptation of GT1 results in augmented phenotypes driven by selection pressures in the extracellular environment. The results demonstrate the complex and polygenic nature of lab adaptation and *in vitro* virulence. We have only scratched the surface of our 300 potentially phenotype-conferring genes and therefore anticipate the discovery of many additional host-independent virulence factors in future work.

## MATERIALS AND METHODS

### *In vitro* culturing of T. gondii.

The GT1 strain of T. gondii was obtained through BEI Resources (catalog no. NR20728) and propagated into culture using ED1 medium (3) supplemented with 10 mM HEPES, pH 7.2. Parasites were maintained in human telomerase reverse transcription (hTERT) immortalized human foreskin fibroblasts (HFFs) in a humidified 37°C incubator under 5% CO_2_. Typically, early-passage GT1 parasites require 3 to 4 days to fully lyse a T25 (25-cm^2^) flask of host cells, while later-passage GT1 parasites (>P80) require 2 to 3 days. Passing was performed serially by transferring 500 μl of the lysed host cell flask (consisting of suspended parasites) into a new T25 flask of hTERT host cells containing 9 ml of warm ED1 medium. Long 1-ml serological pipettes were used for transferring to reduce cross-contamination of separate T. gondii populations. Serial passaging of GT1 occurred in this fashion for up to 220 passages.

After successful establishment of GT1 into culture, several single-cell clones derived from the initial population were obtained. Clonal GT1 populations, named B1, B2, B3, etc., as well as the original polyclonal population, named B0, were frequently frozen down during the ∼220 passages (∼2.5 years) of *in vitro* culturing. Frequent freeze downs of parasite populations ensured a chronologically maintained fossil record of the evolving parasites, a stored resource for future experiments.

### *In vitro* culturing of host cells.

hTERT-immortalized HFF cells were maintained in T175 (175-cm^2^) flasks until P18, when they were passed into T25 flasks by P19 and used as host cells for parasite culture. Primary HFF cells were maintained in T175 (175-cm^2^) flasks until P9, when they were passaged into the desired flasks or plates by P10 and used as hosts for plaque assay or immunofluorescence assay (IFA). Goat skeletal fibroblast (GSF) cells were generously provided by Mahipal Singh of Fort Valley State University (69).

### Plaque assays.

T25 flasks containing medium-to-large vacuoles of parasites (∼2 days postinoculation) were washed twice with 10 ml phosphate-buffered saline (PBS) to remove extracellular parasites and debris, followed by addition of 3 to 6 ml (volume dependent on vacuole size and number) of warm ED1 medium. Next, the cell monolayer was scraped with a rubber police man and the host cells mechanically ruptured by passing through a 27-gauge (27G) needle. Mechanically egressed parasites were filtered through a 3-μm-pore polystyrene filter to remove host cell debris. Parasite concentration was determined with a hemocytometer and adjusted to 10,000 cells/ml in a final volume of 3 ml Ed1 medium.

Three- to 6-week-old primary HFF or GSF monolayers were used for plaque assays. Plaques for GT1 and RH were allowed to form for 11 days on primary HFFs or 14 days of GSF host cells. Host cell monolayers containing plaques were fixed with 100% ethanol for 10 min at room temperature, stained with 5× crystal violet solution for 10 min at room temperature, washed twice with PBS, and allowed to air dry for 24 h. Quantification of plaque size (i.e., *in vitro* virulence) and plaque number relative to input (i.e., invasion efficiency) was performed with FIJI (70).

### Extracellular survival assay.

Parasite cultures were prepared as described above, and 3 ml of the parasite cell suspension was incubated at 37°C and 5% CO_2_ in non-tissue-culture-treated 6-well plates for 0 to 10 h. Plaque assays were performed hourly and quantified as mentioned above. Plaque numbers at each time point were normalized to the 0-h time point to yield percent survival.

### Replication assay.

Mechanically egressed parasites (27G needle) were inoculated onto confluent primary HFF monolayers grown on coverslips in 6-well plates, centrifuged at 1,000 × *g* for 5 min, allowed to invade at 37°C (floating in a water bath) for 10 min, and subsequently washed 3× with PBS. Intracellular parasites were then allowed to replicate for exactly 24 h, followed by 100% methanol fixation and IFA with rabbit α-TgIMC3 (71) to mark the cortical cytoskeleton and 4′,6-diamidino-2-phenylindole (DAPI) to mark DNA. The number of parasites per vacuole was enumerated for 100 vacuoles.

### Egress assay.

Mechanically egressed parasites (27G needle) were inoculated onto 6-well HFF plates containing glass coverslips and allowed to invade and replicate for 30 h. Replacement medium containing either 1 μM A23187, 5% ethanol, or dimethyl sulfoxide (DMSO) was incubated for exactly 5 min in the plates before fixation of infected monolayers with 4% PFA and IFA with α-TgIMC3 and DAPI. The number of egressed vacuoles was enumerated for a total of 50 vacuoles per condition.

### DNA sequencing and analysis.

Parasite genomic DNA was isolated using the Qiagen DNeasy blood and tissue kit (catalog no. 69504) according to the manufacturer’s protocol. Illumina’s library prep kit (FC-121-1030) was used to generate ∼361-bp DNA fragments, on average, which were quantified using a Quibit flex fluorometer (catalog no. Q32851) and quality checked using Agilent’s TapeStation (catalog no. 5067-5584 and 5067-5585). Next, 150-bp paired-end sequencing was performed on Illumina’s NextSeq500 platform using their high-output flow cell kit (FC-404-2004) according to the manufacturer’s protocol. FASTQ reads were then analyzed by RUFUS to call sequence variants between two samples (https://github.com/jandrewrfarrell/RUFUS) (72, 73). To compare RUFUS variants across all samples, all calls were merged and GRAPHITE was used to call genotype across all samples in the study (https://github.com/dillonl/graphite). High-frequency variants were called if the emerging mutation reached 75% allele frequency in at least one evolving population.

### P4-flippase genotyping.

Parasite genomic DNA was isolated using Qiagen DNeasy blood and tissue kit (catalog no. 69504) according to the manufacturer’s protocol. M13 primers (see Table S1, Tab 9, in the supplemental material) were used to PCR amplify the ∼350-bp region surrounding the R270L and A477D alleles. PCR products were purified and sent to Eton Biosciences for Sanger sequencing. Allele confirmation and chromatographs were obtained using 4Peaks (https://nucleobytes.com/4peaks/).

### RNA sequencing and data analysis.

**(i) Library preparation and sequencing.** T. gondii-infected (24 to 36 h) hTERT-immortalized HFF monolayers were washed 3× with PBS and either mechanically lysed with a 27G needle (6 h extracellular) or immediately lysed (intracellular) and processed on ice for RNA isolation using the Qiagen RNeasy kit (catalog no. 74104) according to the manufacturer’s protocol. RNA quality was evaluated by measuring the RNA integrity number (RIN) using Agilent’s TapeStation (kit catalog no. 5067-5579 and 5067-5580). Illumina’s library prep kit (RS-122-2102) was used to generate ∼281-bp cDNA fragments, which were quantified using Qubit flex fluorometer (catalog no. Q32852) and quality checked using Agilent’s TapeStation (catalog no. 5067-5584 and 5067-5585). Next, 75-bp paired-end sequencing was performed on Illumina’s NextSeq500 platform using their high-output (150 cycles) flow cell kit (FC-404-2002) according to the manufacturer’s protocol.

**(ii) Data processing before analysis.** The quality of reads was assessed using FastQC (version 0.10.1). The adapter sequences AGATCGGAAGAGCACACGTCTGAACTCCAGTCA and AGATCGGAAGAGCGTCGTGTAGGGAAAGAGTGT were trimmed from the 3′ ends of the reads with Cutadapt from the Trim Galore package (version 0.3.7) (http://www.bioinformatics.babraham.ac.uk/projects/trim_galore/). The trimmed reads were mapped against the reference genome of T. gondii, GT1 (ToxoDB release 41), and assembled with HISAT2 (version 2.0.5) (20). The overall alignment rate was above 90% for 6-h extracellular samples and between 25% and 60% for intracellular samples. SAM files obtained from alignment results were processed using SAMtools (version 1.4.1), and the relative abundance of transcripts was estimated using featureCounts (21). Counts per million (CPM) values per gene were quantified using the cpm() function from the edgeR Bioconductor R package (version 3.24.3) (21). Genes with CPM value of >2 in at least 3 samples were retained for further analysis. Gene counts were normalized and scaled to logarithmic form using edgeR’s TMM method (trimmed mean of *M* values) with DGEList(), calcNormFactors(), and cpm() functions. The cpm() parameters were y = DGEList.obj, log=TRUE, prior.count = 3, and normalized.lib.sizes=TRUE. Batch effects were examined and visualized by hierarchical clustering using the R function hclust() with the default parameters and log CPM expression values. Hierarchical clustering classified some samples in a single batch that were relatively far from their associated biological replicates based on Euclidean distance metric. This was also observed in the MDS plots (multidimensional scaling plot of distances between gene expression profiles). The plotMDS() function from edgeR was utilized to generate the plot and visualize outliers. Batch correction was performed using removeBatchEffect() from the Limma R package with the following parameters: x = logCPMexpr, batch=batch, design=design to correct for unknown technical batch effects and avoid their ramification on downstream analysis.

**(iii) DEA.** DEA was carried out using edgeR. A design matrix was generated with the model.matrix() function for the treatments/conditions (13 factors) and batches to perform pairwise comparisons. A normalized DEGList object was constructed from counts and treatments with DGEList() and calcNormFactors(). The estimateDisp() function was then used to estimate the dispersion based on Cox-Reid profile-adjusted likelihood (CR). The estimateDisp() parameters were y = DGEList.obj, design = design.matrix, and robust=TRUE. The quasi-likelihood negative binomial generalized log-linear model was then fitted to the count data by glmQLFit() with robust parameter set to TRUE. The returned object of class DGEGLM from glmQLFit() was passed to glmQLTest() to ascertain the DEGs. The most DEGs ranked either by FDR-adjusted *P* value (*q* value) or by absolute log FC [abs(logFC)] were extracted with the function topTags(). Transcripts with 2-fold and higher differences in their expression levels [abs(logFC) > 1] and *q* value of <0.05 were considered significant DEGs.

**(iv) Clustering analysis.** Temporal patterns of the DEGs were captured using R package TCseq (version 1.12.1) (26); tca() function was used to generate a tca object for the time course temporal analysis. Tca() function requires 3 inputs, including experiment design, genomicFeature (gtf) file, and counts table. The experiment design was generated from RNA sequencing information, with columns having the sample identifiers (IDs) and time points. The GT1 annotation file (gtf format) with the gene’s ID and location was used for the genomicFeature parameter. Since tca() only accepts the raw integer counts (not normalized expression), we used the MDS plots and hierarchical clustering to identify the noisy samples (replicates) and removed them from the raw count data. Biological replicates from passages 35 and 210 were excluded from the temporal analysis. Once tca.obj was constructed, Dbanalysis() quantified the log FC values using tca.obj and by fitting the negative binomial generalized linear model to the read counts. The normalized time course table containing expression values of all extracellular samples was created with timecourseTable() function with the parameters tca = tca.obj, value = “expression,” and filter = FALSE. Clustering of time course data was done in an unsupervised manner using the Cmeans (CM) method as implemented in TCseq in timeclust(). The total number of clusters was set to 8 (trial and error). Detected patterns were standardized and visualized. To label the detected clusters as being either strongly up or down trending, a linear regression model was fitted to the data in each cluster. The clusters with a large positive or negative slope of the regressed line and *R*^2^ of >0.5 were selected as trending clusters, resulting in two up-trending and one down-trending cluster.

**(v) Linear mix-effect models with regression splines.** Regression analysis (RA) was performed to identify the genes that demonstrate strong correlation with evolution of GT1’s phenotypic traits over passages. The phenotype measurements (plaque size, invasion efficiency, and extracellular survival) were collected at 9 time points (P12, P33, P51, …, P223) with ≥3 replicates at each time point. Three biological replicates of RNA-Seq data were collected at seven passages (P7, P11, P35, P55, P85, P148, and P210) and four passages (P7, P11, P85, and P148) for extracellular and intracellular parasites, respectively. A mixed-effect regression spline model was fitted to the phenotypic and RNA-Seq time course data separately using the following mixed-effect model:

$$y_{i}\left(t_{j}\right)=\mu \left(t_{j}\right)+\gamma_{i}\left(t_{j}\right)+\epsilon_{\mathrm{ij}}$$

where μ(t) is the fix effect corresponding to population mean, $\gamma_{i}\left(t\right)$ stands for the random effect corresponding to deviation of each gene (phenotype) from mean at each time point, and $\epsilon_{\mathrm{ij}}$ is the assumed independent normally distributed noise. The random effect is assumed to be generated from a Gaussian process, $\gamma_{i}∼\mathrm{GP}\left(0,\delta \right)$, with mean of 0, which implies that the measured values of $\gamma_{i}\left(t_{j}\right)$ are normally distributed with covariance matrix $D\left(\ell ,\;s\right)=\delta \left(t_{\ell },t_{s}\right)$. The mixed-effect model was applied through its implementation in sme() function from the R package sme (74) with time course data and criteria = AIC. The mean curve was calculated for each gene (phenotype) by sme(). Since the time points at which the RNA-seq and phenotype data were collected were not aligned, we generated a custom script to fit a natural cubic spline to the returned coefficients from sme(). Using the fitted spline, the missing values corresponding to gene expression (phenotypes) were predicted at several time points spanning the common range of passages. Next, we calculated the Spearman and Pearson correlation between the fitted means of all genes and all phenotypes using the R function cor().

The fitted object returned by the combination of sme() and spline() models was used to visualize the mean curve along with the confidence bands at 95% level. The variability around the mean curve was derived from the variance-covariance matrix of the fitted model quantified with vcov() R function given the fitted object.

**(vi) GLASSO TF analysis.** We assembled a PPI network involving annotated T. gondii GT1 TFs comprising all annotated ApiAP2 domain-containing proteins (including an unnamed ApiAP2 on chromosome IX, TGGT1_215895, which we named for the next available numeric, AP2IX-10, and seven Myb domain-containing proteins, TGGT1_200385 [BFD1], TGGT1_203950, TGGT1_213890, TGGT1_264120 [Myb1], TGGT1_275480, TGGT1_306320 [Myb2], and TGGT1_321450 [Myb3]) by applying a Gaussian graphical model (GMM) to our RNA-Seq data. The GMM captures the direct pairwise relationships between the nodes in the interaction graph by estimating the covariance and the precision matrix ${\text{Θ}}^{-1}$ from the sample covariance matrix *S*. Each AP2 and Myb represents a node in the graph, and the edges represent a direct interaction between them after accounting for partial correlations. The objective function of the GGM is given by

$$\underset{\text{Θ}}{\max}\log(\det\text{Θ})-\mathrm{tr}\left(S\text{Θ}\right)-\text{Λ}{\left|\left|\text{Θ}\right|\right|}_{1},$$

where *S*, Θ, and Λ are the empirical covariance, precision matrix, and penalty matrices, respectively.

The normalized expression values (CPM) across all the samples and conditions were used to determine the empirical covariance matrix. Estimation of the sparse inverse covariance matrix with *L*_1_ regularization was performed with the GlassoFast package (75). For selecting the optimal regularization parameter, Λ, a path of parameters from 0.1 to 1 with 0.1 step size was used, and the Kullback–Leibler divergence was calculated. The best Λ was chosen as the value where the second derivative of the $\mathrm{KL}({\text{Θ}}_{{\text{Λ}}_{j}},{\text{Θ}}_{{\text{Λ}}_{j+1}})$ function was smaller than a constant (76). Once the optimal Λ was selected, the inverse covariance matrix was estimated accordingly. Strong associations between AP2s were identified as those with absolute value of partial correlation greater than 0.01. The Igraph R package (V1.2.5) was used to visualize the network.

**(vii) Ranking gene candidates.** Genes were ranked based on the following criteria: (i) significant correlation (0.7) with at least one phenotype (∼3,000 genes); (ii) differentially expressed in at least one passage compared with baseline P11 (986 genes); and (iii) trending (up or down, 439 genes). This resulted in a list of 300 genes.

**(viii) Software code.** Code is available on GitHub through https://github.com/umbibio/ToxoplasmaGondii.

### Plasmids and parasite strain generation.

All oligonucleotides used are provided in Table S1, Tab 9. Synthesized and annealed forward and reverse oligonucleotides, serving as single guide RNAs, were cloned into BsaI-digested pU6-Universal plasmid (Addgene number 52694) to generate our final CRISPR/Cas9 plasmids (77). A DHFR selection cassette was amplified with 60-bp primers to yield a 2,700-bp repair template containing the entire 5′-untranslated region (UTR), 3′UTR, and coding DNA sequence of DHFR, along with 39-bp arms complementary to the site of Cas9-mediated DSBs (double-strand breaks) within the gene of interest (GOI). To knock out a GOI, 20 μg of each CRISPR/Cas9 plasmid was cotransfected with 20 μg of the DHFR selection cassette to enable DSB and homology-directed repair at the locus. Successful homologous recombination of the DHFR selection cassette into the GOI locus was confirmed with diagnostic PCR primer sets. Successful ablation of mRNA expression was confirmed by quantitative RT-PCR. In short, RNA was extracted with the Qiagen RNeasy kit, cDNA transcribed with SuperScript III reverse transcriptase (Thermor-Fisher), and qPCR performed on a QuantStudio system (Applied Biosystems) using the B1 gene, encoding glycerol-3-phosphate dehydrogenase (78), as a control and signals quantified by the ΔΔ*C_T_* method (79).

### Life stage score analysis.

Previously published tachyzoite, merozoite, bradyzoite, and sporozoite RNA-Seq data sets were used to generate stage scores (23, 80); all RNA-Seq data sets were obtained from ToxoDB.org and downloaded as TGME49 gene IDs, which were then converted into TGGT1 gene IDs using the “syntenic orthologs” tool on ToxoDB.org. This process excluded 496 TGGT1 gene IDs from the analysis. Four singular time points from these available data sets were chosen for DE analysis (“tachyzoites,” “tissue cysts,” “Mero 3,” and “sporozoite day 4”). For each gene, all three possible differential expression scenarios between these four data sets were calculated. For each gene, the number of ≥2-fold upregulated scenarios were enumerated and considered the stage score (score = 0 to 3). To validate the established stage scores, three previously published gene sets were examined for significant enrichment in their indicated life stage (25). For analysis of our RNA-Seq data sets, the stage score of our upregulated and downregulated gene sets were individually calculated. In an alternative approach, we defined representative genes for each life stage by only selecting genes with a stage score of 3 (469 of the 989 genes in the extracellular samples). To examine clustering of life-stage-specific genes, PCA was applied to mean log CPM expression values of the 496 stage-specific genes in extracellular samples. We utilized the built-in R function prcomp() with parameters data = logCPM and scale = TRUE to attain the first two principal components for further visualization.

### Bootstrap analysis.

To empirically estimate the statistical significance of the scores, we performed bootstrap analysis as follows. For each gene set, 1,000 random genes were sampled with replacement, and the mean scores of all four life stages were calculated. The distribution of the mean scores for each life stage were estimated from the bootstrap samples, and the *P* value of the observed mean score of each gene set was calculated by summing over the right tail of the distribution.

### Enrichment analyses.

Previously published gene sets (25) along with tachyzoite data were utilized for GSEA. Fisher’s exact test was used to assess the statistical significance of the overlaps between differentially expressed genes in various contrasts, trending genes, and classes derived from our data and the gene sets. The R function fisher.test() calculated *P* values corresponding to the overlaps between two gene sets. The fisher.test() parameters were x = contingency.table and alternative = “greater.” A *P* value of ≤0.05 was used as cutoff for significance. Moreover, *P* values were adjusted with the function p.adjust() with method “BH.” GOEA and MPEA were performed using the “Analyze results” feature on ToxoDB.org (81).

### Protein modeling.

Protein Homology/Analogy Recognition Engine v2.0 (Phyre2) prediction and analysis tools (http://www.sbg.bio.ic.ac.uk/~phyre2/html/page.cgi?id=index) were used. Subsequently, we predicted the impact of the SNPs on the structure using Missense3D predictive structural analysis (http://missense3d.bc.ic.ac.uk/missense3d/) (17).

### Lipidomic analysis.

Lipid analysis followed previously published methods (82). Specifically, extracellular parasites were harvested by 3-μm filtration and pelleted at 1,000 × *g* for 20 min, washed three times with 5 ml PBS, resuspended in 1 ml 100% methanol, and stored at 4°C. Lipids were extracted by adding 0.5 ml methanol, vortexing, and adding 5 ml methyl-*tert-*butyl ether (MBTE; Sigma-Millipore number 650560). Samples were immediately shaken for 1 h at 800 rpm at room temperature. Phase separation was induced by adding 1.25 ml deionized water and 10 min of RT incubation and 10 min of centrifugation at 1,000 × *g*. The lower phase was reextracted with 2 ml of fresh upper-phase solution (prepared from a blank sample). Samples were dried under nitrogen gas and resuspended in dichloromethane before analysis by a reflectron time-of-flight mass spectrometer (AccTOF; JEOL USA Inc., Peabody, MA) equipped with a DART ion source (DART-SVP; IonSense Inc., Saugus, MA). Standards of C_14:0_ myristic acid (M3128; Sigma) and C_16:0_ palmitic acid (P0500; Sigma) were used as controls. Mass spectra were measured in negative-ion mode at a resolving power of >10,000 (full width at half maximum) and a spectral acquisition mass range of *m/z* 100 to *m/z* 1,000. The atmospheric pressure interface potentials were the following: orifice 1, 20 V; ring lens, 5 V. The ion guide potential was set to 900 V to permit detection of ions with *m/z* greater than ∼90. Fomblin Y (Sigma-Aldrich) was used for external mass calibration. Mass spectrometry absolute intensity peaks corresponding to specific fatty acid species (C_14:0_, C_16:0_, C_18:0_, C_18:1_, and C_19:0_) were converted into ratios (medium, C_14:0_ and C_16:0_) to total species and C_16:0_ to long-chain (C_18:0_, C_18:1_, and C_19:0_) species. Mean values and standard errors were plotted using the R package ggplot2. Individual experimental groups (low passage, high passage, and RH) were then subjected to a Shapiro-Wilk test for normal distribution and a Levene’s test for equal variance (R, car package). Once assumptions were validated, a two-sided unpaired Student's *t* test (car) with equal variances was conducted between groups.

### Mutation rate calculation.

T. gondii’s mutation rate was determined with the following formula: mutation rate = mutations/base pairs/generation. The variables were the following: mutations, the total number of mutations that accumulated for each B2-B86 clone; base pairs, the T. gondii genome is 63,950,00 bp (ToxoDB v49); generations, because T. gondii viability increases with lab adaptation, the population doubling level (PDL = log [harvested/seeded]/log_2_; as described previously [83]) decreases with time. To account for the dynamic PDL during lab adaptation, the PDL was calculated at several time points (P16, P39, P95, P150, and P265) and plotted as a function of time (Fig. S8a). The total number of generations over the course of the experiment was estimated by calculating the area under this curve, resulting in 520 generations.

### Statistical analysis.

A Student’s two-tailed equal variance *t* test was used to determine the significance (*P* value) of evolved samples compared to the lowest-passage-number sample. Adjusted *P* values (*q* values) were calculated using the Benjamin-Hochberg method as the FDR. For stage score analysis, both a Student’s two-tailed *t* test and an independent bootstrap analysis (*n* = 1,000 random sampling) were used to determine significance. A one-sided Fisher’s exact test was used to quantify the significance (*P* value) of each gene set’s enrichment in the database.

### Data availability.

Short-read DNA-Seq and RNA-Seq data were deposited as FASTQ data to the NCBI Sequence Read Archive (SRA) under BioProject number PRJNA782463.
