# Supplementary figures and images for: The Extracellular Milieu of Toxoplasma's Lytic Cycle Drives Lab Adaptation, Primarily by Transcriptional Reprogramming
Source: mSystems. 2021 Dec 7;6(6):e01196-21. doi: 10.1128/mSystems.01196-21 (PMC8651083; doi:10.1128/mSystems.01196-21)

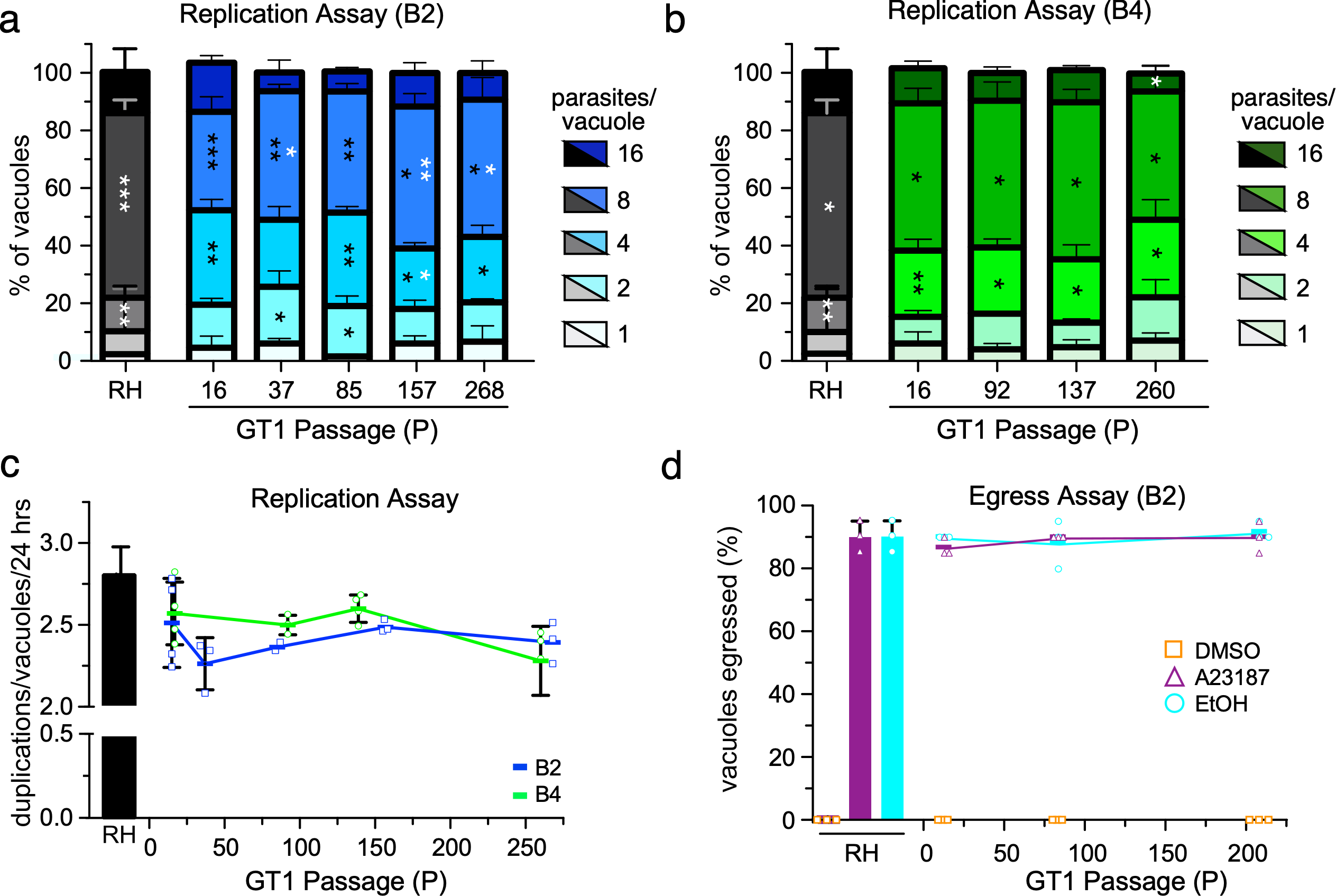

Supplement: FIG S1 [file msystems.01196-21-sf001.tif]

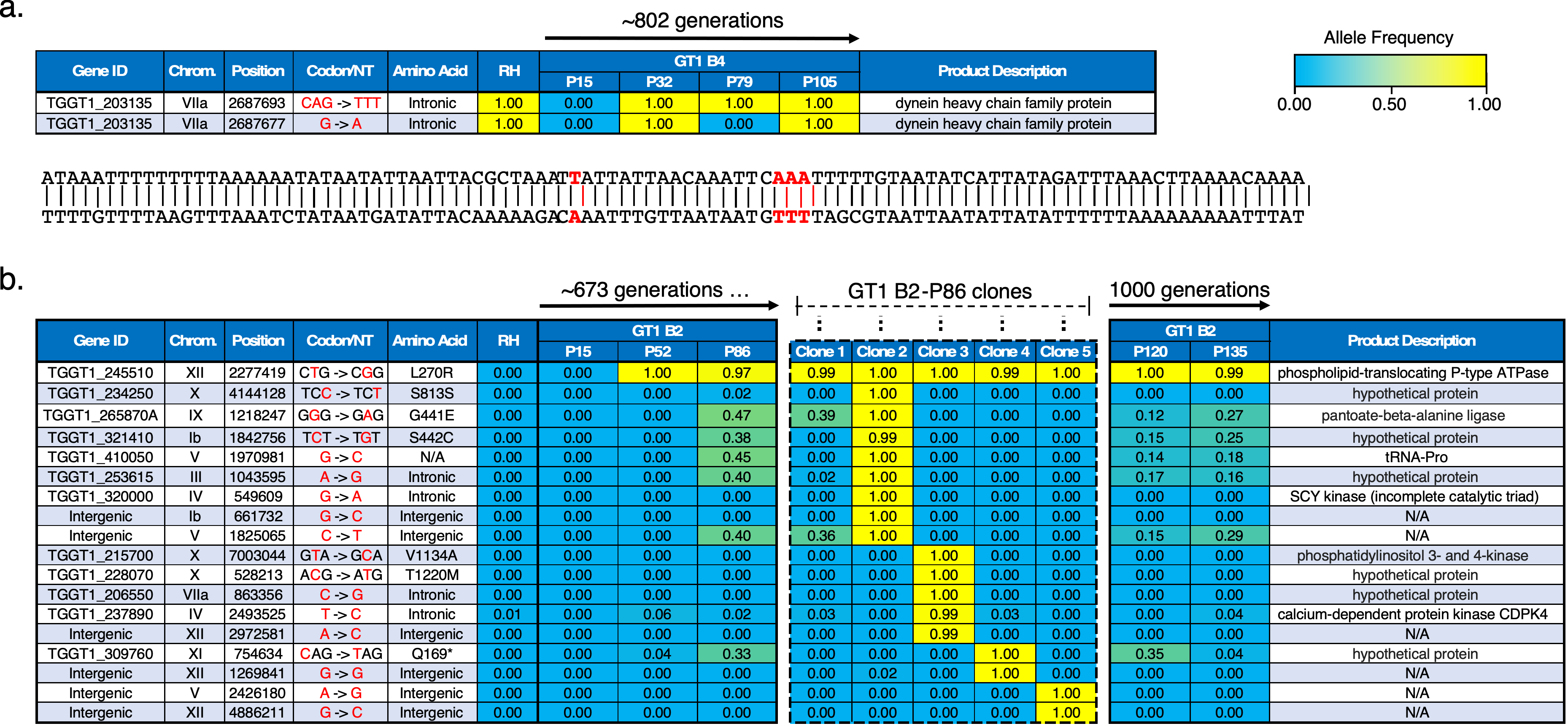

Supplement: FIG S2 [file msystems.01196-21-sf002.tif]

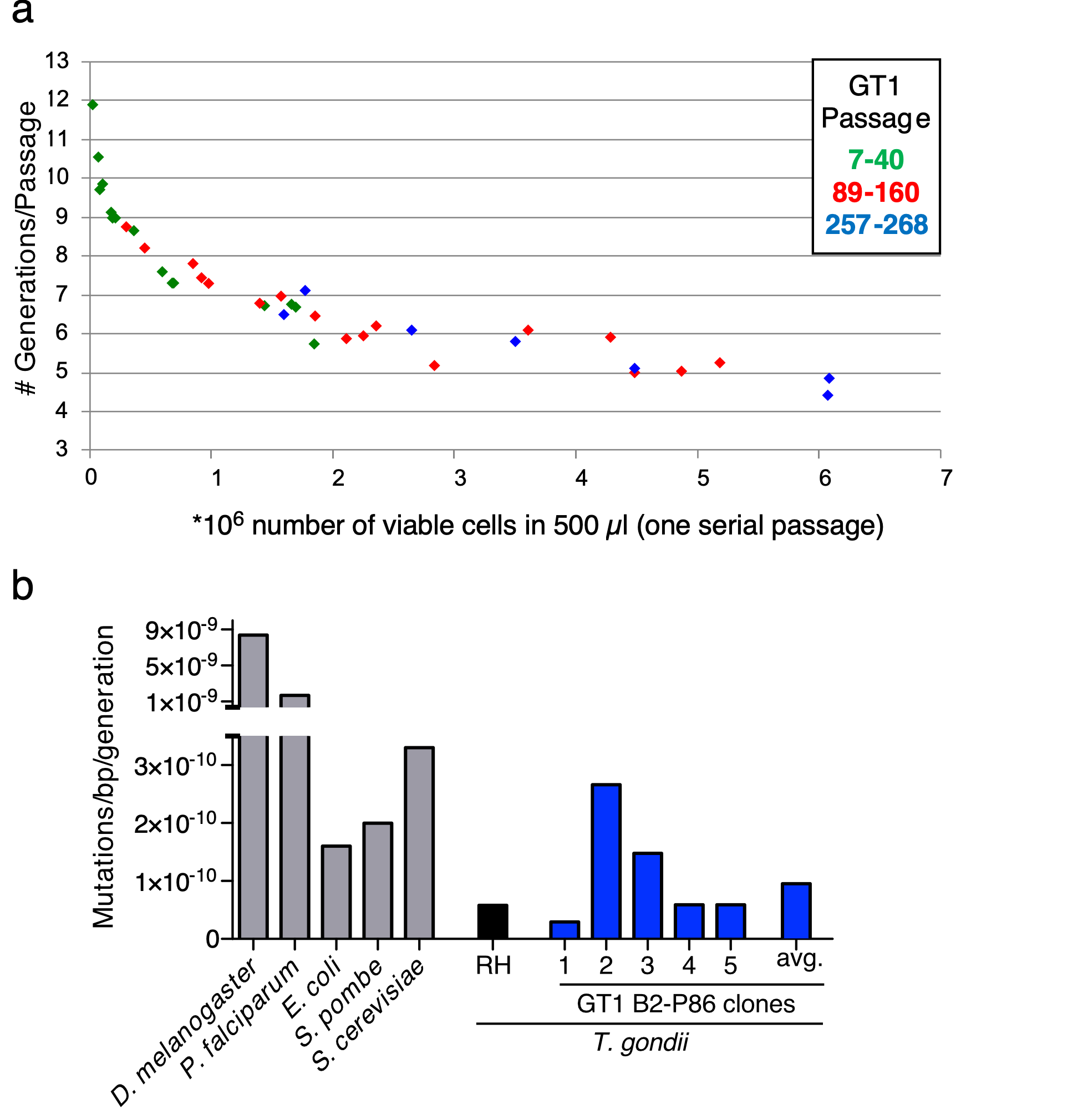

Supplement: FIG S3 [file msystems.01196-21-sf003.tif]

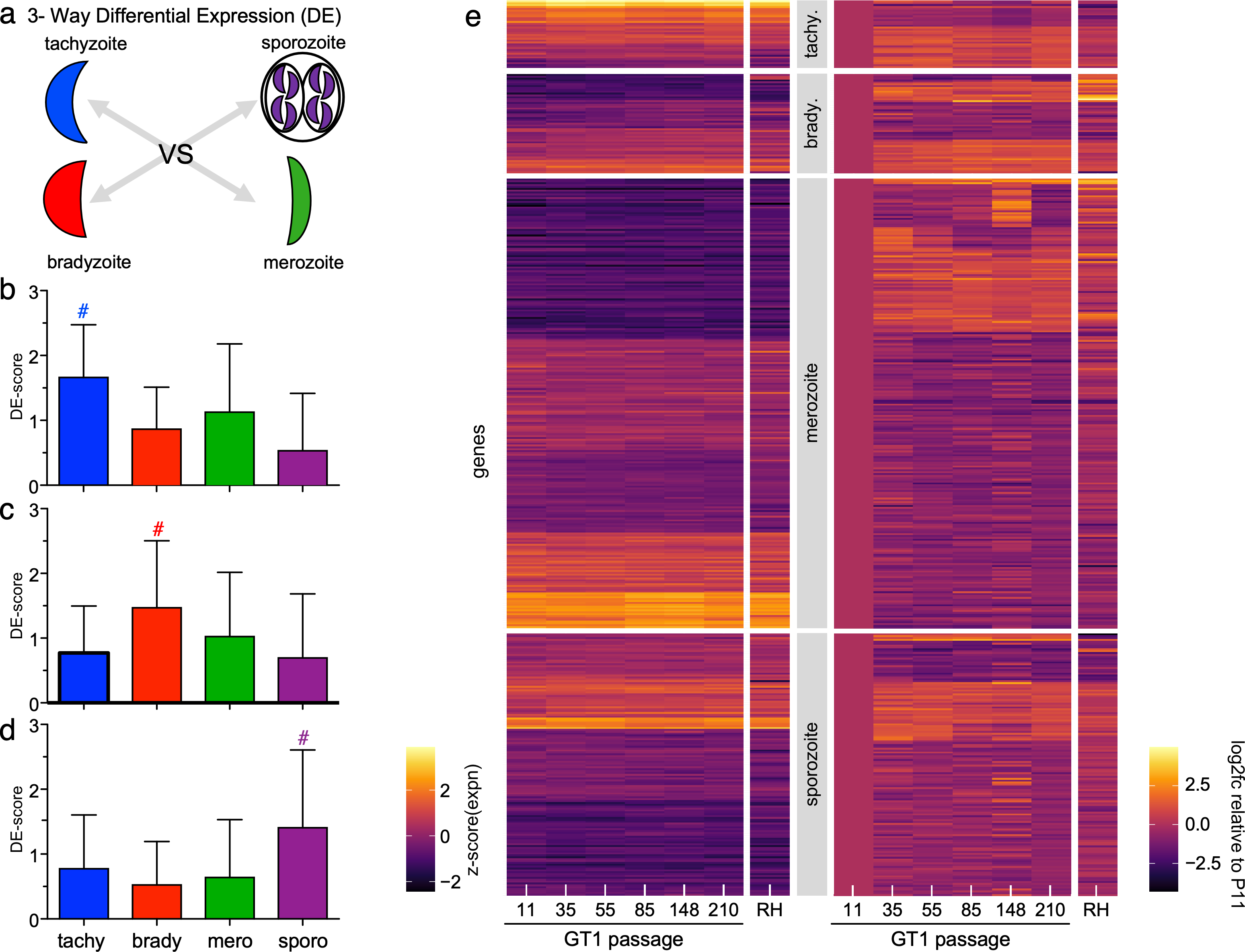

Supplement: FIG S4 [file msystems.01196-21-sf004.tif]

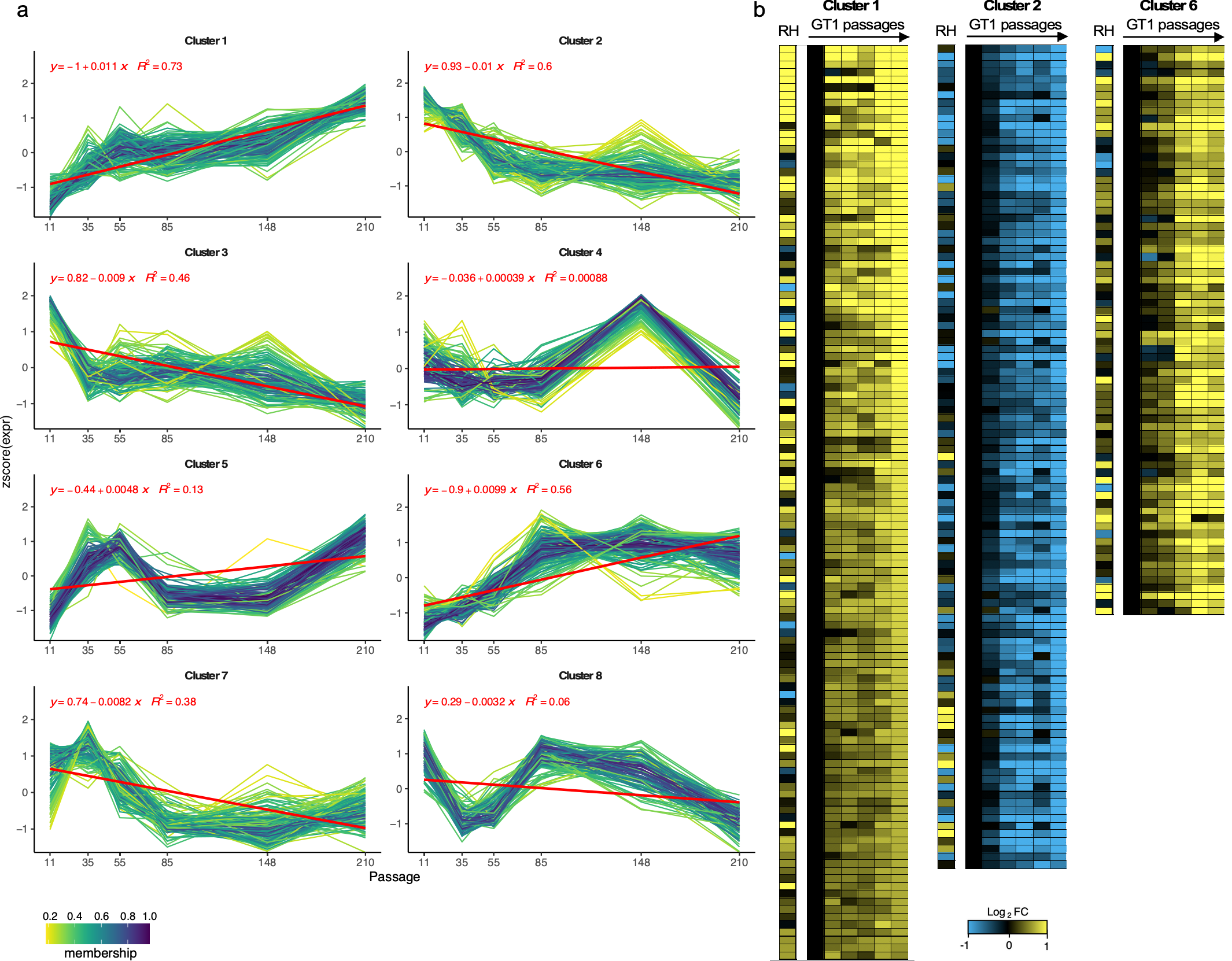

Supplement: FIG S5 [file msystems.01196-21-sf005.tif]

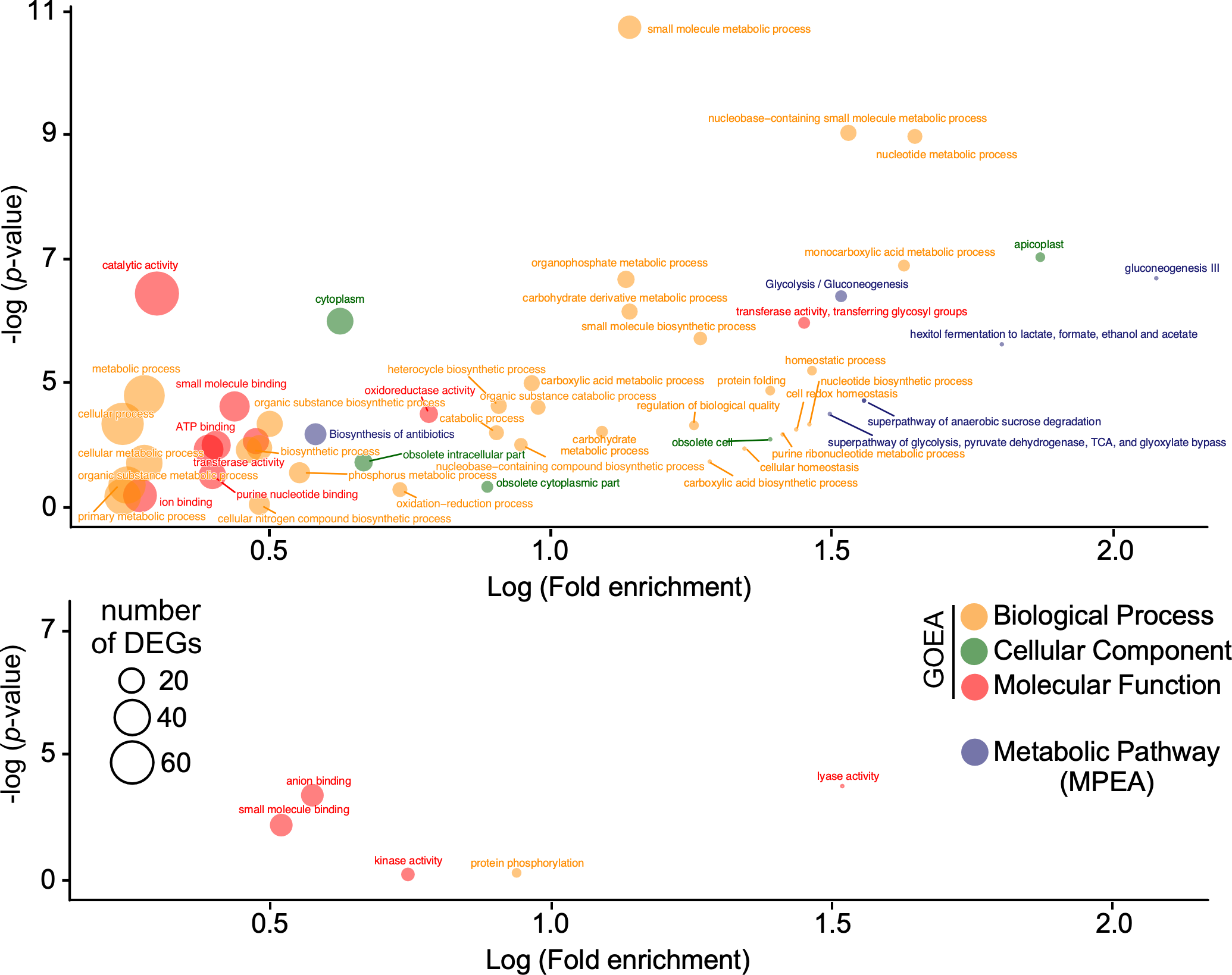

Supplement: FIG S6 [file msystems.01196-21-sf006.tif]

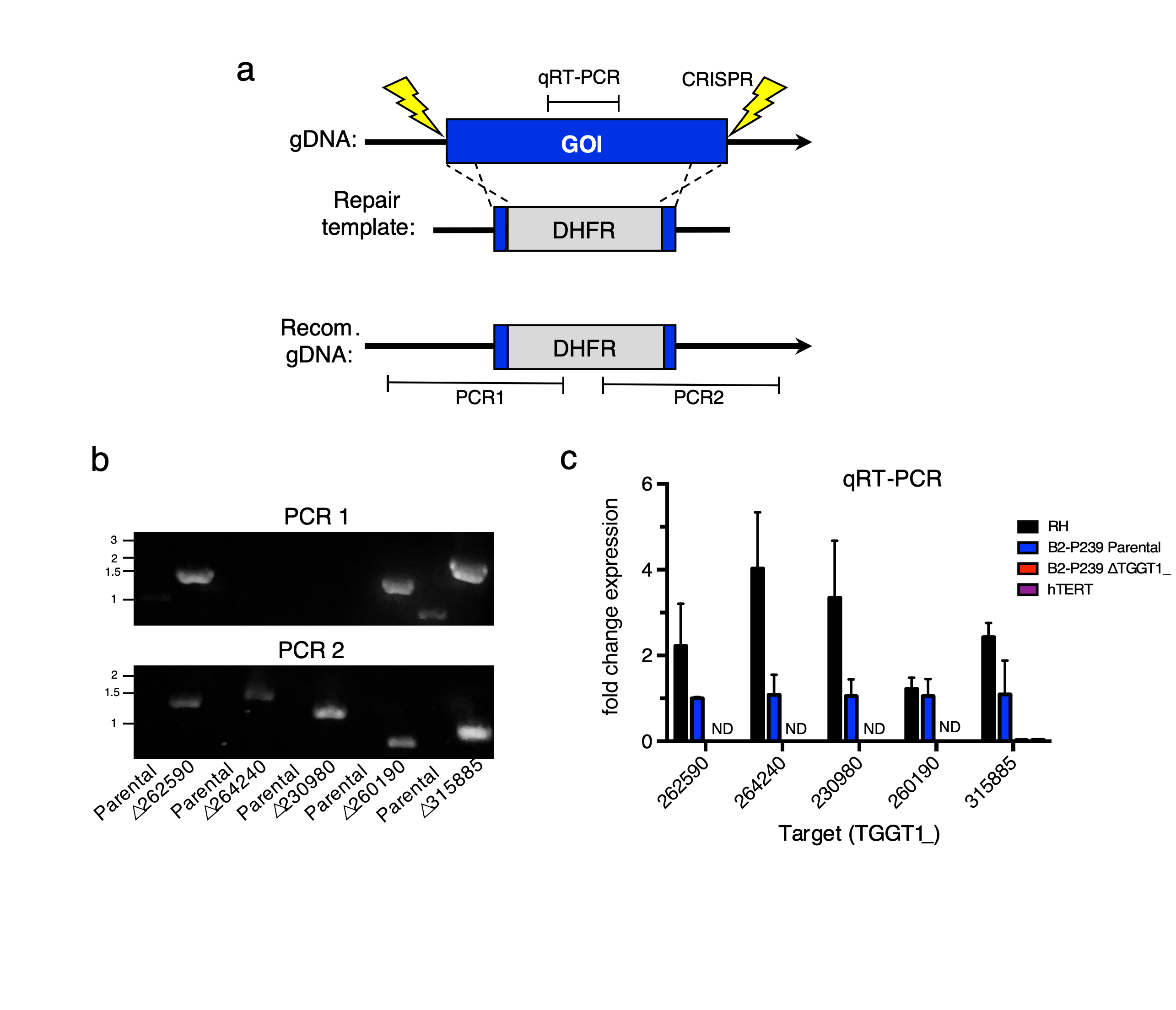

Supplement: FIG S7 [file msystems.01196-21-sf007.tif]

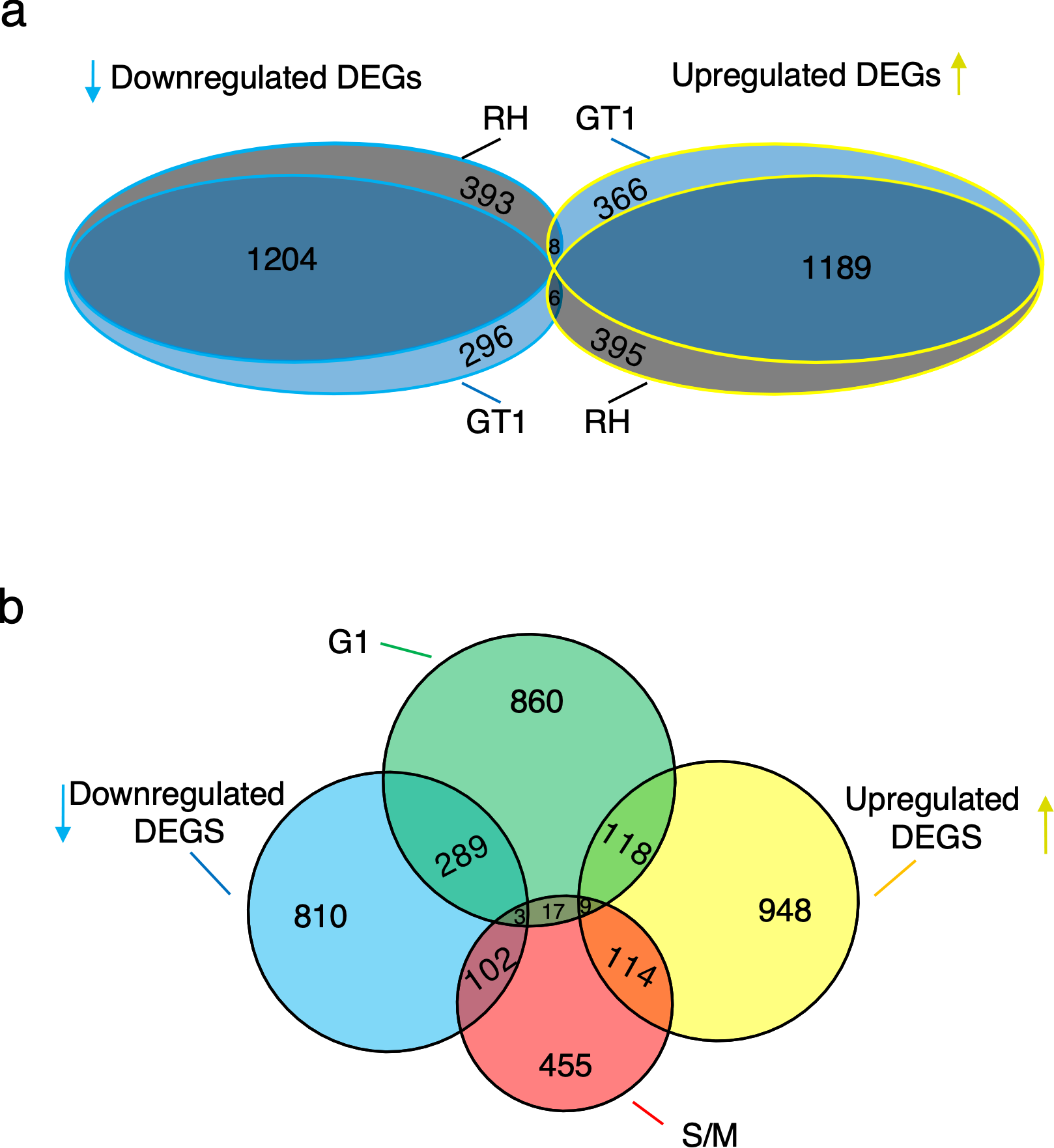

Supplement: FIG S8 [file msystems.01196-21-sf008.tif]
